# Immune Infiltrating Cells-Derived Risk Signature Based on Large-scale Analysis Defines Immune Landscape and Predicts Immunotherapy Responses in Glioma Tumor Microenvironment

**DOI:** 10.3389/fimmu.2021.691811

**Published:** 2021-08-13

**Authors:** Nan Zhang, Hao Zhang, Zeyu Wang, Ziyu Dai, Xun Zhang, Quan Cheng, Zhixiong Liu

**Affiliations:** ^1^Department of Neurosurgery, Xiangya Hospital, Central South University, Changsha, China; ^2^One-Third Lab, College of Bioinformatics Science and Technology, Harbin Medical University, Harbin, China; ^3^Department of Clinical Pharmacology, Xiangya Hospital, Central South University, Changsha, China; ^4^National Clinical Research Center for Geriatric Disorders, Xiangya Hospital, Central South University, Changsha, China

**Keywords:** tumor microenvironment, gliomas, immunotherapy, somatic mutation, immune checkpoint, machine learning

## Abstract

The glioma tumor microenvironment (TME), composed of several noncancerous cells and biomolecules is known for its complexity of cancer-immune system interaction. Given that, novel risk signature is required for predicting glioma patient responses to immunotherapy. In this study, we systematically evaluated the TME infiltration pattern of 2877 glioma samples. TME phenotypes were determined using the Partitioning Around Medoid method. Machine learning including SVM-RFE and Principal component analysis (PCA) were used to construct a TME scoring system. A total of 857 glioma samples from four datasets were used for external validation of the TME-score. The correlation of TME phenotypes and TME-scores with diverse clinicopathologic characteristics, genomic features, and immunotherapeutic efficacy in glioma patients was determined. Immunohistochemistry staining for the M2 macrophage marker *CD68* and *CD163*, mast cell marker CD117, neutrophil marker CD66b, and RNA sequencing of glioma samples from the XYNS cohort were performed. Two distinct TME phenotypes were identified. High TME-score correlated with a high number of immune infiltrating cells, elevated expression of immune checkpoints, increased mutation rates of oncogenes, and poor survival of glioma patients. Moreover, high TME-score exhibited remarkable association with multiple immunomodulators that could potentially mediate immune escape of cancer. Thus, the TME-score showed the potential to predict the efficacy of anti-*PD-1* immunotherapy. Univariate and multivariate analyses demonstrated the TME-score to be a valuable prognostic biomarker for gliomas. Our study demonstrated that TME could potentially influence immunotherapy efficacy in melanoma patients whereas its role in immunotherapy of glioma patients remains unknown. Therefore, a better understanding of the TME landscape in gliomas would promote the development of novel immunotherapy strategies against glioma.

## Highlights

- The TME-score comprehensively evaluate the infiltration characteristics of the TME cells in glioma patients.
- The TME-score is an independent prognostic biomarker to predict patients’ survival.
- TME-score showed the potential to predict the efficacy of anti-*PD-1* immunotherapy.

## Introduction

According to the 2016 World Health Organization (WHO) classification criteria, gliomas are classified into low-grade glioma (LGG) and glioblastoma (GBM). Gliomas are the most common and devastating primary tumors affecting the central nervous system (1). The prognosis of GBM patients is dismal and the median overall survival (OS) is about 15 months following concomitant chemoradiotherapy, which can be attributed to the excessive heterogeneity of GBMs, rendering traditional therapies ineffective.

Immune checkpoint blockers such as *PD-1/L1* and *CTLA-4* have demonstrated remarkable clinical efficacy in the management of multiple cancers (2, 3). However, the current checkpoint immunotherapy is only effective in a limited number of glioma patients. It is, therefore, important to develop more effective immunotherapies for gliomas.

Besides genetic and epigenetic variations in glioma cells, tumor microenvironment (TME) also plays a critical role in tumor proliferation, progression, and therapeutic responses (4, 5). TME is a complex network of cancer cells, stromal cells and, most importantly, infiltrating immune cells. The TME complexity makes it difficult to predict the immunotherapy outcome in gliomas effectively. Cancer cells regulate numerous biological functions through direct or indirect interaction with TME components (6). Emerging evidence suggests that TME crucially influences the response to both chemotherapy (7) and immunotherapy (8). Moreover, alterations in the number of immune infiltrating cells in the TME have been shown to affect clinical outcomes in various malignant tumor types. Therefore, it is important to characterize the TME landscape in gliomas.

Understanding the complexity of the TME landscape in gliomas may lead to the identification of different immune-related TME phenotypes. This can help guide and predict immunotherapeutic responses and reveal potential therapeutic targets. Bioinformatics analysis has been used to evaluate the abundance of immune infiltrating cells in the TME. Several studies have also explored how TME affects immunotherapeutic response and other clinical outcomes (9, 10).

In this study, we developed a novel TME scoring system to improve the clinical management of glioma patients based on large-scale samples.

## Materials and Methods

### Glioma Datasets and Preprocessing

The following search terms were used as: (((survival OR prognosis OR prognostic OR outcome OR death OR relapse OR recurrence))) AND (Glioma[Title]) OR (Astrocytoma*[Title]) OR (Glioblastoma*[Title]) OR (Ependymoma*[Title]) OR (Oligodendroglioma*[Title]) OR (Gliosarcoma*[Title]) OR (Astroglioma*[Title]) OR (LGG[Title]) OR (HGG[Title]) OR (glial cell tumor[Title]). All datasets were manually examined. Patients lacking survival information were excluded from further evaluation.

Publicly available glioma gene-expression datasets together with clinical annotations were downloaded and examined. 2877 samples from 12 patient cohorts diagnosed with gliomas were included in this study (**Table S1**). Four external datasets were included for validation: GSE13041, GSE16011, GSE61335, GSE68838. The microarray datasets were downloaded from the Gene-Expression Omnibus (GEO; https://www.ncbi.nlm.nih.gov/geo/). Chinese Glioma Genome Atlas (CGGA) datasets were downloaded from the CGGA website (http://www.cgga.org.cn/), while The Cancer Genome Atlas (TCGA) datasets were downloaded from UCSC Xena (https://xenabrowser.net/).

Raw data from microarray datasets were generated using Affymetrix and Agilent. The RMA algorithm was used to perform quantile normalization and background correction of the raw data from Affymetrix in the Affy software package. The final summarizing of oligonucleotides for each transcript was based on the consensus median polish algorithm in the Affymetrix software. The raw data from Agilent was processed using limma software. RNA-sequencing data were downloaded from the TCGA and CGGA data portals and the fragments per kilobase million (FPKM) values were transformed into transcripts per kilobase million (TPM) values, which were similar to those resulting from microarrays and comparable between samples. The TPM values from TCGA and CGGA had similar signal intensity with the RMA-standardized values from microarray datasets. R package sva was then used to remove the computational batch effect.

### Estimation of TME-Infiltrating Cells

The CIBERSORT algorithm was used to predict the presence and quantify the abundance of immune cells in glioma samples (11). The LM22 gene signature was applied since it allowed for sensitive and specific discrimination of 22 human infiltrating immune cell phenotypes. Gene-expression profiles were uploaded to the CIBERSORT web portal (http://cibersort.stanford.edu/). This algorithm was run using the LM22 signature and 1000 permutations. Single factor analysis was performed on the 22 immune cells to determine their prognostic values in gliomas. The cellular correlation among the 22 immune cells was performed using Pearson correlation analysis. TIMER algorithm (9), EPIC algorithm (12), MCPcounter algorithm (13), quanTlseq algorithm (14), xCell algorithm (15), and ssGSEA algorithm (16) were also used for estimating the abundance of immune infiltrating cells.

### Unsupervised Consensus Clustering for TME-Infiltrating Cells

Tumors with qualitatively diverse TME infiltrating patterns were classified using Partitioning Around Medoid (PAM) (17), which identified TME patterns and grouped patients for further analysis. The optimal number of clusters and their stability and reliability in the meta-cohort and TCGA cohort were determined using the ConsensuClusterPlus R package. Infiltration level of stromal cells and immune cells in glioma samples was assessed using the consensus ESTIMATE (Estimation of Stromal and Immune cells in Malignant Tumor tissues using Expression) algorithm (18).

### Identification of TME-Related Differentially Expressed Genes (DEGs)

To identify genes associated with TME cell-infiltrating patterns, the patients were grouped into two distinct TME clusters based on the diverse expression of infiltrating immune cells. The enrichment levels of immune signatures were quantified by the xCell algorithm to validate TME clusters (15). The R package limma (19) was used to determine DEGs associated with the two TME cell-infiltrating patterns. The adjusted P-value < 0.01 was used to determine DEGs among the TME subtypes.

### Generation of TME Gene Signatures and Dimension Reduction

The DEGs among the TME clusters were standardized in all samples in the TCGA glioma cohort. Univariate cox regression analysis identified representative DEGs. The unsupervised clustering method (20) was used to classify patients into two TME gene clusters for further analysis. The clusterProfiler R package (21) was used to annotate the TME pattern genes. The consensus clustering algorithm (22) was performed to define the gene clusters. χ^2^ contingency test was used to determine the correlation between the TME gene clusters. The SVM-RFE algorithm was used for dimension reduction and to mitigate the interference effect of redundant genes (23). The top 300 DEGs between two TME gene clusters were selected (24), among which 63 most representative genes were identified with the highest accuracy of separating samples. Principal component analysis (PCA) was performed and the extracted principal component 1 served as the signature score. A method similar to GGI was then applied (25) to define the TME-score of each patient after the prognostic value of gene signature score was obtained:

TME−score=ΣPC1i −ΣPC1j

where i is the signature score of clusters with HR>1, and j represents the expression of genes with HR<1.

### Pathway Enrichment Analysis

All gene sets were downloaded from the MSigDB database. Gene set enrichment analysis (GSEA) and gene set variation analysis (GSVA) were performed on the TME gene signatures using the clusterProfiler R package and GSVA R package (21). Pathways enriched in TME immune-related gene patterns were identified in Gene Ontology (GO) and Kyoto Encyclopedia of Genes and Genomes (KEGG) with the false discovery rate (FDR) < 0.05 and a strict cutoff of P < 0.01.

### Prediction of Immunotherapy Response

The Tumor Immune Dysfunction and Exclusion (TIDE) algorithm was used to infer individual responses to immunotherapy such as immune checkpoint blockade (*e.g.* anti-*PD-1* therapy) (26). The submap analysis was applied to compare differences in response to anti-*PD-1* and anti-*CTLA-4* therapies. For the melanoma data set (GSE78220, N=28), GSE78220 expression profiles (FPKM normalized) were transformed into TPM values, which were used to calculate the TME-score (27). T cell-inflamed gene expression profile (GEP) was defined through the expression of the 18 genes (28). Cytotoxic activity (CYT) was determined based on the gene expression value of two cytolytic markers (GZMA and PRF1) (29), and the geometric mean of these two markers was used to perform the calculations. Seven types of immune checkpoints were collected from previously published work (30).

### RNA Sequencing

For each glioma patient of the 48 samples, major exclusion criteria were incomplete follow-up data, poor quality of samples, and missing baseline clinicopathological features. RNAstore-fixed tumor tissues were then collected for sequencing. Briefly, 1 μg RNA per sample was used as input material for RNA sample preparations. RNA was extracted and sheared followed by sequencing library preparation using NEBNext^®^ UltraTM RNA Library Prep Kit. Subsequently, PCR was performed with Phusion High-Fidelity RNA polymerase, Universal PCR primers and the Index (X) Primer. After PCR primer removal, biotin-labeled probe was used for capturing target regions. The captured libraries were sequenced on an Illumina Hiseq platform and 125 bp/150 bp paired-end reads were generated. Raw data (raw reads) of fastq format were first processed through in-house perlscripts. In this step, clean data (clean reads) were obtained by removing reads containing adapter, ploy-N, and low-quality reads from raw data. At the same time, Q20, Q30, and GC content of the clean data were calculated. All downstream analyses were based on clean data with high quality. Reference genome and gene model annotation files were downloaded from the genome website directly. The reference genome index was built using Hisat2 v2.0.5 and paired-end clean reads were aligned to the reference genome using Hisat2 v2.0.5 and Hisat2 was selected as the mapping tool. FeatureCounts v1.5.0-p3 was then used to count the reads numbers mapped to each gene. TPM of each gene was calculated based on the gene length and reads count mapped to this gene.

### Immunohistochemistry Staining

Immunohistochemistry (IHC) staining was conducted as previously described (31, 32). Paraffin-embedded tissues of 40 glioma samples with the corresponding sequencing data from the Xiangya Neurosurgey (XYNS) cohort were used for performing IHC. The paraffin-embedded glioma sections were incubated with *CD68*, *CD163, CD117* (Rabbit, 1:500, Proteintech, China), and CD66b (Rabbit, 1:200, Abcam). The IHC marker was detected with microscope.

### Statistical Analysis

The normality of variables was tested using the Shapiro-Wilk normality test (33). For normally distributed variables, unpaired Student’s t-test was used to compare differences between two groups, while the Wilcoxon test was used to compare nonnormally distributed variables. For multiple groups, one-way analysis of variance was used as a parametric method to compare mean values between groups while Kruskal–Wallis tests were used as a nonparametric method.

Pearson analysis and distance correlation analyses were used to calculate correlation coefficients. Contingency tables were analyzed by χ^2^ contingency test. The OS and TME-score were calculated using the R package survival and cutoff values determined. Based on the dichotomized TME-score, patients were grouped into high or low TME-score in each data set, and the computational batch effect was reduced by the R package sva. Data were mainly visualized using the R package ggplot2. R package, igraph was used to visualize the cellular interactions within the TME. For the differential gene expression analysis, we used the Benjamini–Hochberg method that converts the P values to FDRs to identify significant genes (34). The package pROC (35) was utilized to establish receiver operating characteristic (ROC) curves and calculate the area under the curve (AUC). OncoPrint was used to delineate the mutation landscape of TCGA *via* the maftools R package (36). The Kaplan–Meier method was applied to generate and visualize survival curves for the subgroups, and the differences between data sets were compared using the log-rank test. The univariate and multivariate Cox proportional hazards regression models were utilized to calculate the hazard ratios in univariate and multivariate analyses and to determine independent prognostic factors using the R package survival. All survivorship curves were generated *via* R package survminer. All heatmaps were generated based on pheatmap. All statistical analyses were performed in R version 3.6.1 (https://www.r-project.org/). All tests were two-sided and P values < 0.05 were considered to be statistically significant.

## Results

### The Landscape of Glioma TME

**Figure 1:**
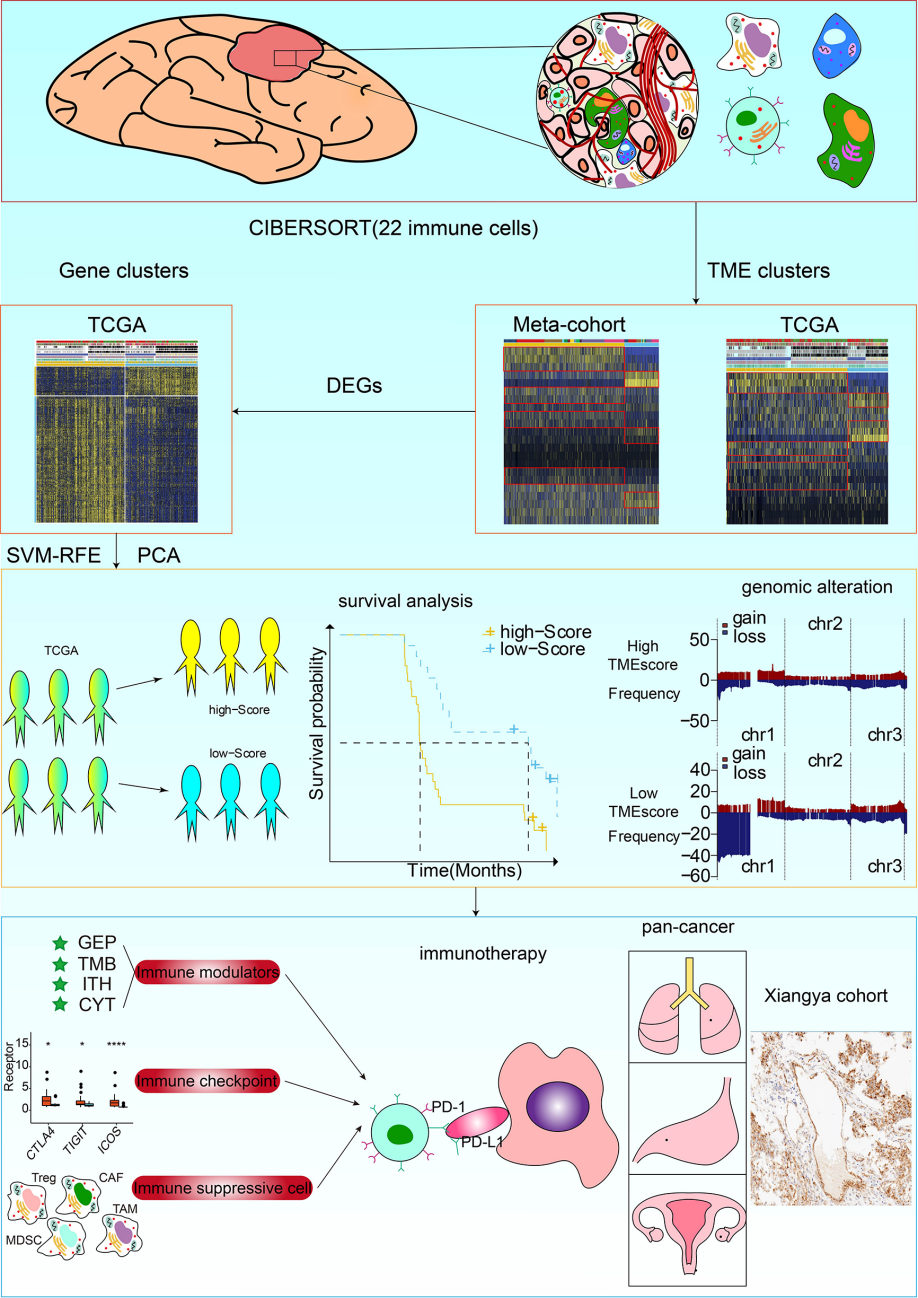
Flow diagram of the study design. TME phenotypes were determined using the Partitioning Around Medoid method. Principal component analysis (PCA) was used to construct a TME scoring system. A total of 857 glioma samples from four datasets were used for external validation of the TME-score. The correlation of TME phenotypes and TME-scores with diverse clinicopathologic characteristics, genomic features, and immunotherapeutic efficacy in glioma patients was determined. Immunohistochemistry staining for the M2 macrophage marker *CD68* and *CD163*, and RNA sequencing of glioma samples from the XYNS cohort were performed.

**Figure 2:**
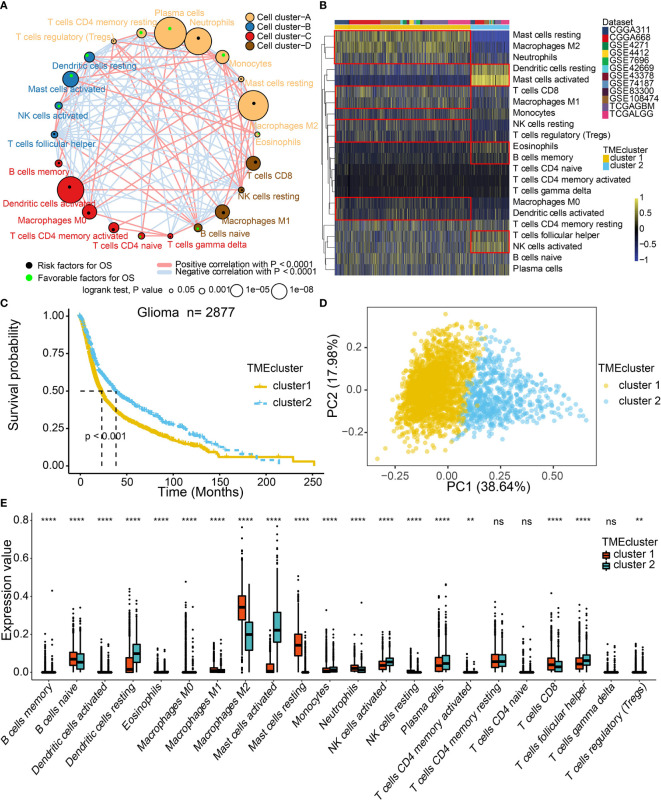
TME landscape in gliomas and characteristics of TME subtypes in the meta-cohort. **(A)** Cellular interaction of the TME cell types. **(B)** Unsupervised clustering of TME cells for 2877 patients in the meta-cohort. **(C)** Kaplan–Meier curves for two TME groups of 2877 patients in the meta-cohort. Log-rank test, P < 0.001. **(D)** PCA separated the two TME clusters. **(E)** Fraction of TME cells in two TME clusters. The scattered dots represent TME cell expression values within each group. NS, Not Statistically Significant; **P < 0.01; ****P < 0.0001.

The TME cell infiltration patterns and gene signatures were evaluated (**Figure 1**). The ConsensusClusterPlus package was used to assess clustering stability to determine the optimal cluster number (**Figure S1A**), which supported two robust subtypes of gliomas in a meta-cohort. The optimal cluster number was also identified in TCGA (**Figure S1B**). We built an integrated TME cell network that comprehensively depicted a landscape of TME cell lineages, tumor immune cell correlations, and their prognostic values on the OS of glioma patients (**Figure 2A** and **Tables S3, S4**). A similar TME cell network was constructed in the TCGA cohort (**Figure S2A** and **Tables S3, S4**). Partitioning Around Medoid (PAM) was performed for the 2877 tumors with the corresponding TME cell expression profiles of the 12 included glioma cohorts (**Figure 2B** and **Table S2**). PAM was subsequently performed in the TCGA cohort (1027 patient samples), and two phenotypes were separated by different clinical factors (**Figure S2B** and **Table S6**). Two TME phenotypes were identified by TME cell infiltration and exhibited significant differences in the OS in the meta-cohort and TCGA alone (log-rank test, p < 0.001; **Figures 2C** and **S2C**). Among the two clusters, PCA distribution was separated in both the meta-cohort and TCGA alone (**Figures 2D** and **S2D**). **Figure 2E** shows that the two TME clusters exhibited significant differences in TME cell infiltration patterns, and these differences were reported in multiple immune suppressive cell types. Additionally, PAM was performed in another two cohorts for validation, and there was a significant correlation between identified TME phenotypes and TME infiltration cell patterns in the CGGA and GSE108474 datasets, respectively (**Figures S2E, F** and **Table S6**). We further analyzed immune cells by the xCell algorithm (15). The two TME clusters identified by xCell algorithm were significantly different in terms of the survival probability (**Figure S3E**). Additionally, the TME clusters identified by xCell algorithm were consistent with those identified by the CIBERSORT algorithm (**Figure S3F**).

### Functional Annotations for TME Phenotype Clusters

To elucidate the correlation between the immune infiltrating environment and TME clusters, 20 of both immune-related and DNA regulation-related signaling pathways in GO analysis were identified in the meta-cohort (**Figure S3A**). We found that TME cluster1 was associated with immunosuppressive pathways (**Figure S3A**). Additionally, TME cluster 1 was associated with pathways regulating tumor cell proliferation (**Figure S3A**). Similar results were observed in the TCGA database (**Figure S3B**), showing differences in 20 signaling pathways in the two TME clusters (**Figures S3C, D**).

### Generation of TME Gene Signatures and Functional Annotation

We acquired a total of 1312 DEGs (**Table S5**) using the limma package to classify the patients into genomic subtypes and to investigate the potential biological characteristics of each TME infiltration cell pattern. The analysis was significantly consistent with the clustering results of the TME phenotype groups (χ^2^ contingency tests, p =1.95 × 10^-12^). The TCGA glioma cohort population was grouped into two TME gene clusters 1 and 2 (**Figure S4A**). The survival analysis of the two patient clusters indicated that gene cluster 1 correlated with worse survival outcomes than cluster 2 (**Figure S4B**). The GO and KEGG enrichment analyses showed that gene clusters 1 and 2 were enriched in distinct biological processes. In GO enrichment analysis, gene cluster 1 was involved in tumor proliferation (**Figure S4C**). Overexpression of genes involved in immune activation pathways was enriched in gene cluster 2 (**Figure S4D**). Additionally, the KEGG enrichment analysis showed that gene cluster 1 was associated with tumor proliferation and was a prognostic marker for poor survival outcomes (**Figure S4E**). Gene cluster 2 was associated with immune activation and was a prognostic marker for better survival outcomes (**Figure S4F**).

### Generation of TME-Score, Transcriptome Traits, and Clinical Characteristics

The SVM-RFE algorithm was used in dimension reduction to extract phenotype signatures with high classification accuracy and further explore the role of TME phenotypes. Sixty-three most representative DEGs were identified (**Figure S5A** and **Table S7**); the chromosomal distribution and expression of these genes are displayed in **Figure S5B**. Almost all of the 63 genes were significantly differentially expressed between glioma molecular subtypes, isocitric dehydrogenase (IDH) mut glioma and IDH wt glioma (**Table S13**). The regulatory networks identified by the clusterProfiler R package suggested that immune activation and tumor proliferation pathways were involved and exhibited significant overlaps with other pathways (**Figure S5C**).

**Figure 3:**
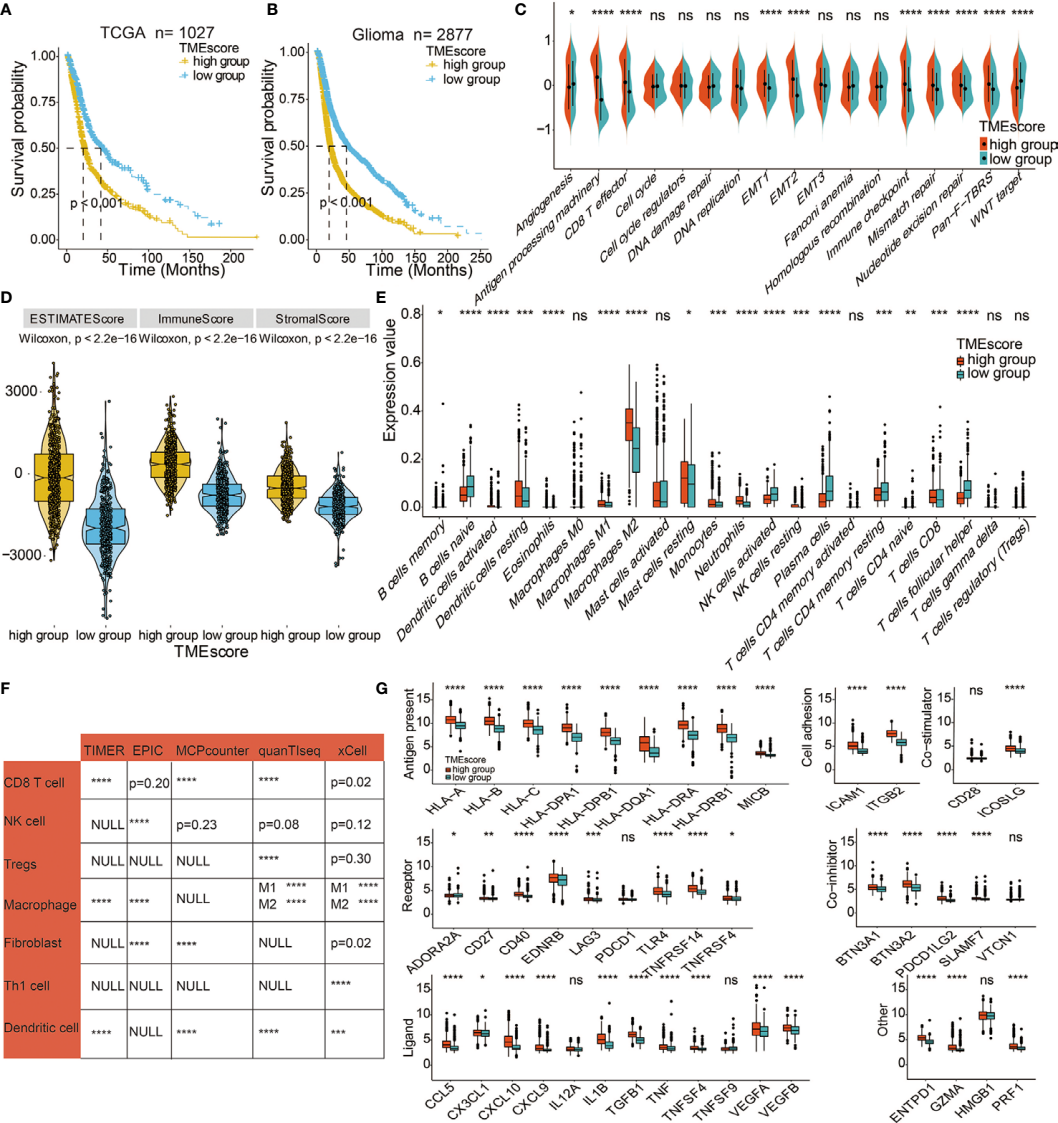
Immune-related characteristics of the TME score. **(A)** Kaplan–Meier curves for high and low TME-score patient groups in TCGA. Log-rank test, P < 0.001. **(B)** Kaplan–Meier curves for the high and low TME-score patient groups in the meta-cohort. Log-rank test, P < 0.001. **(C)** TME-score patient groups were distinguished by different known signatures (immune, mismatch, and stromal signatures as indicated) in TCGA. The scattered dots represent the mean value of signature genes within each group. **(D)** Expression difference of Estimate Score, Immune Score, and Stromal Score in TME-score in TCGA. **(E)** Fraction of TME cells in TME-score in TCGA. Scattered dots represent TME cell expression values. **(F)** Correlation between TME-score and TME cells calculated by different algorithms in TCGA. **(G)** Fraction of seven types of immune checkpoints in TME-score in TCGA. Scattered dots represent immune checkpoint expression values. NS, Not Statistically Significant; *P < 0.05; **P < 0.01; ***P < 0.001; ****P < 0.0001.

The PCA algorithm was used to define the TME-scores of the 12 cohorts (**Table S8**). Based on the 63 DEGs, PCA distribution was separated among the two TME gene clusters in TCGA (**Figure S5F**). The interconnections among TME clusters, TME gene clusters, TME-scores, patient survival, and tumor grade are presented in **Figure S5D**. Contingency table revealed the significant consistency between TME clusters and TME-scores, which TME score could be considered a collection of the features of the two TME clusters (**Figure S5E**). The distribution of TME-scores in TME clusters in CGGA and GSE108474 datasets are shown in **Figure S5G**. Consistent with the findings in TME gene cluster 1, a high TME-score was a prognostic marker for poor clinical outcomes in TCGA and meta-cohort (**Figures 3A, B**). Given that gliomas consist of various types and grades of glial tumors, the differences in TME landscapes among different types of glial tumors were explored. The prognostic values of TME-scores were verified in TCGA LGG (**Figure S6A**) and TCGA GBM (**Figure S6B**). The TME-score was also a prognostic marker for the IDH status in LGG (**Figure S6C**), GBM (**Figure S6D**), and pan-gliomas (**Figure S6E**). A high TME-score was associated with metastatic and immunosuppressive signatures (**Figure 3C**). The correlations between TME-scores and these known signatures are shown in **Figure S5H**.

The associations between TME-scores and the immune infiltrating environment was further examined. High TME-scores were correlated with Estimate Scores, Immune Scores, and Stromal Scores (**Figure 3D**) and also associated with the infiltration of M2 macrophages, mast cells, and neutrophils. Thus, high TME-scores were an indication of the immunosuppressive environment and poor survival outcomes while low TME-scores were prognostic for activated immune environments (**Figures 3E**, **S5I**). Further, TME-scores were significantly correlated with CD8 T cell, NK cell, regulatory T cells (Tregs), macrophages, fibroblasts, Th1 cells, and dendritic cells based on TIMER algorithm, EPIC algorithm, MCPcounter algorithm, quanTlseq algorithm, and xCell algorithm (**Figure 3F**). The immunocyte infiltrating characteristics of TME-scores were verified in LGG (**Figure S6F**) and GBM (**Figure S6G**). Gliomas with a high TME-score expressed more immune checkpoints, such as LAG3, CD40, and PDCD1LG2 (**Figure 3G**). **Figure S7B** displays the expression differences of TME-scores in relation to several clinical factors. Gliomas with unmethylated MGMT, IDH WT, 1p19q non-codeletion, higher grade, and mesenchymal gliomas with poor survival outcomes had high TME-scores.

### TME-Score Is Associated With Unique Genomic Alteration Patterns

**Figure 4:**
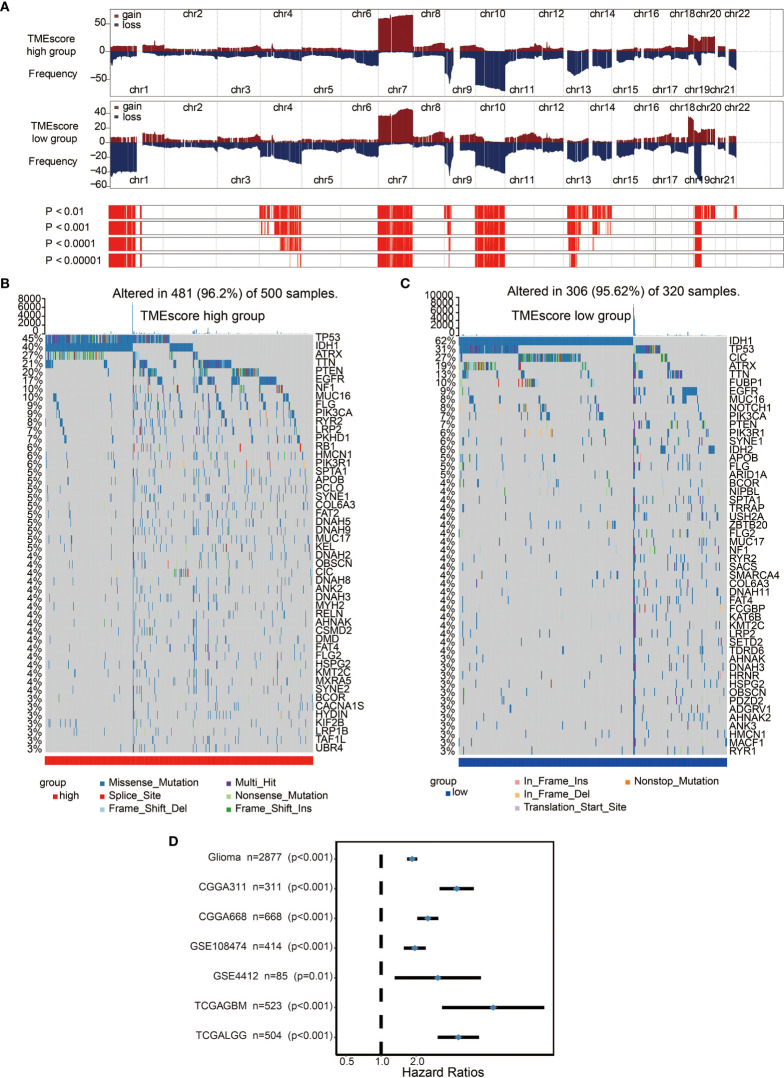
Distinct genomic profiles associated with the TME-score. **(A)** GISTIC 2.0 amplifications and deletions in gliomas with high and low TME-scores. Chromosomal locations of peaks of significantly recurring focal amplifications (red) and deletions (blue) are presented. **(B)** Differential somatic mutations were detected in gliomas with high TME-score. **(C)** Differential somatic mutations were detected in gliomas with low TME-score. **(D)** Subgroup analyses estimating the clinical prognostic value between low/high TME-score groups in independent glioma datasets.

We performed a copy number variant (CNV) (**Figure 4A**) and somatic mutation analysis (**Figures 4B, C**) of the TCGA dataset to determine the associations between TME-score and glioma genomic profiles. Various frequently amplified and deleted genomic regions were observed in high and low TME-score samples (**Figure 4A**). The somatic mutation analysis showed mutation frequencies of various genes as follows: *TP53* (45%), *IDH1* (40%), *ATRX* (27%), *TTN* (21%), and *EGFR* (20%) in the high TME-score (**Figure 4B**), while *IDH1* (62%), *TP53* (31%), and *CIC* (27%) in the low TME-score cluster (**Figure 4C**). A CNV comparison between high and low TME-score clusters identified significantly different CNV regions (**Table S11**). The mutated genes were compared between high and low TME-score clusters, identifying different mutated genes (**Table S12**).

### Potential Intrinsic Immune Escape Mechanisms of TME-Score

**Figure 5:**
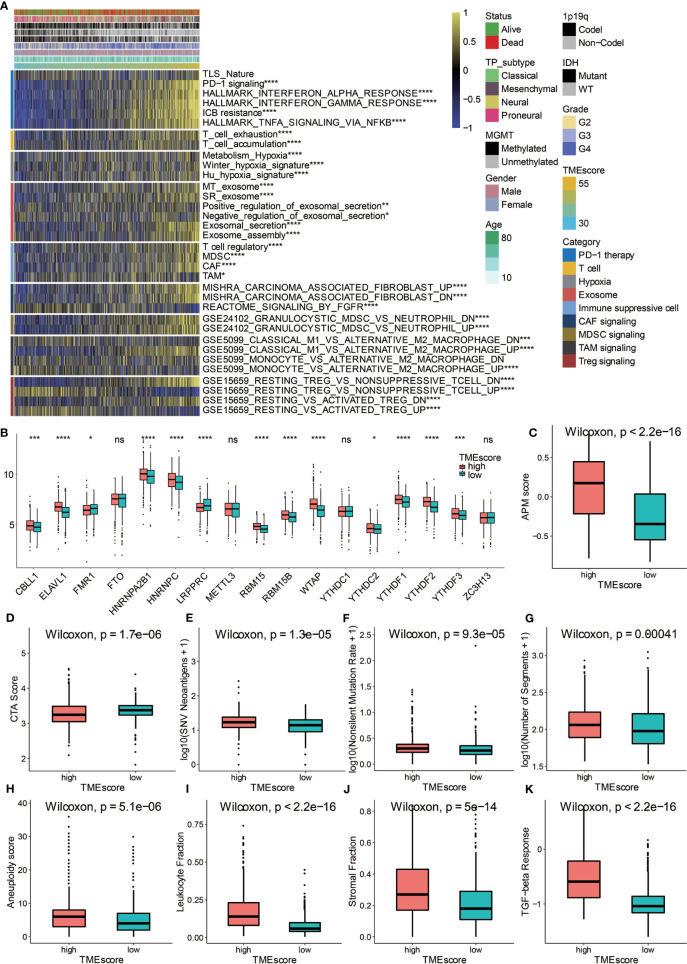
Potential immune escape mechanisms related to TME-score. **(A)** Characterization of the immune suppressive signatures associated with TME-scores in TCGA. **(B)** Fraction of m6A signature genes in TME-score in TCGA. **(C)** APM score in high and low TME-score. **(D)** CTA score in high and low TME-score. **(E)** SNV neoantigens in high and low TME-score. **(F)** Nonsilent mutation rate in high and low TME-score. **(G)** Number of segments in high and low TME-score. **(H)** Aneuploidy score in high and low TME-score. **(I)** Leukocyte fraction in high and low TME-score. **(J)** Stromal fraction in high and low TME-score. **(K)** TGF-beta response in high and low TME-score. NS, Not Statistically Significant; *P < 0.05; **P < 0.01; ***P < 0.001; ****P < 0.0001.

**Figure 6:**
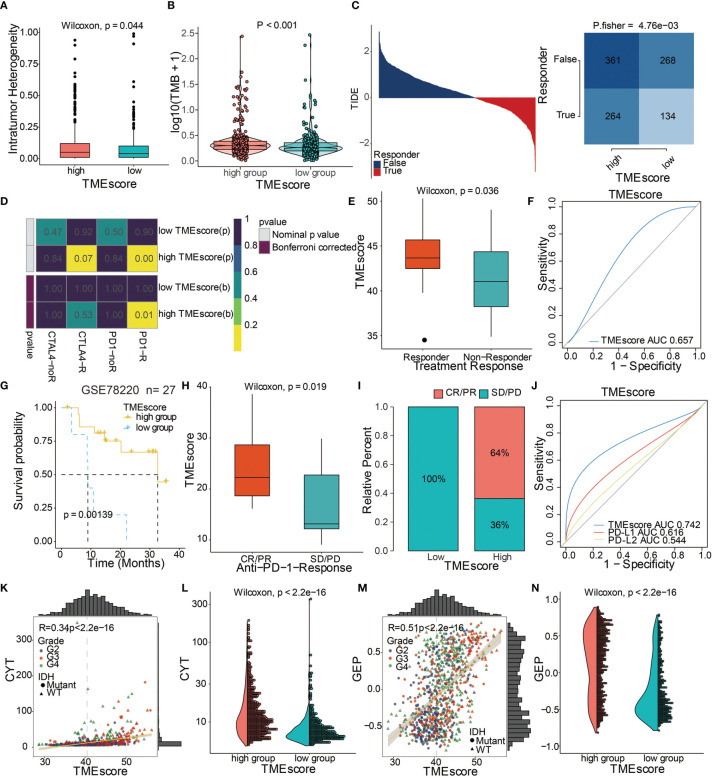
TME-score is a prognostic biomarker and predicts immunotherapeutic benefit. **(A)** Intratumor heterogeneity in high and low TME-score. **(B)** TMB in high and low TME-score. **(C)** TIDE value and response to immunotherapy of patients with TME-scores. **(D)** Submap analysis based on the TIDE algorithm showed a significant difference in response to *CTLA-4* and anti-*PD-1* therapy with respect to the TME-score in TCGA. **(E)** TME-scores in groups with a response and non-response to anti–*PD-1*. Differences between groups were compared by the Wilcoxon test (Wilcoxon, P = 0.036). **(F)** Predictive value of the TME-score measured by ROC curves in the GSE35640 cohort. AUC is 0.657. **(G)** Kaplan–Meier curves for high and low TME-score patient groups in the GSE78220 cohort. Log-rank test, P = 0.00139. **(H)** TME-scores in groups with different anti–*PD-1* clinical response status (CR/PR and SD/PD). Differences between groups were compared by Wilcoxon test (Wilcoxon, P = 0.019). **(I)** Rate of clinical response (CR/PR, SD/PD) to anti–*PD-1* immunotherapy in high or low TME-score groups in the GSE78220 cohort. **(J)** Predictive value of the TME-score, *PD-L1, and PD-L2* measured by ROC curves in the GSE78220 cohort. AUC is 0.742. Scatter plots depicting a positive correlation between TME-score and **(K)** CYT and **(M)** GEP. Pearson Correlation Coefficient R = 0.34 and 0.51, respectively. **(L)** CYT and **(N)** GEP expression differences in high and low TME-scores. Differences between groups were compared through the Wilcoxon test (Wilcoxon, P < 0.001).

High TME-scores demonstrated significant enrichment of PD-1 signaling, T cell signaling, Hypoxia signaling, exosome signaling, immunosuppressive cells including Tregs, myeloid-derived suppressor cells (MDSCs), tumor-associated macrophages (TAMs), and cancer-associated fibroblasts (CAFs) (**Figure 5A**). m6A signatures that were associated with antitumor immunity were also enriched in high TME-scores (**Figure 5B**). In term of antigen presentation capacity, high TME-scores presented higher antigen processing and presenting machinery (APM) score (**Figure 5C**). Cancer testis antigen (CTA) and neoantigens were vital source of tumor-specific antigens, and they were significantly higher in high TME-scores (**Figures 5D, E**). A series of factors associated with tumor immunogenicity was then assessed. High TME-scores presented higher level of nonsilent mutation rate, number of segments, and aneuploidy score, all of which were significant indicators for genome alteration (**Figures 5F–H**). Stroma signatures including TGF-beta response, leukocyte fraction, and stromal fraction were higher in high TME-scores (**Figures 5I–K**). Intratumor heterogeneity and tumor mutation burden (TMB) predicting better immunotherapy responses were also higher in high TME-scores (**Figures 6A, B**).

### The TME-Score Predicts Therapeutic Benefits

We assessed the TME-scores in glioma cohorts because of their prognostic significance associated with poor outcomes in glioma datasets (**Figure 4D**). Survival analysis in the 10 included cohorts indicated an association of the high TME-score with poor survival outcomes in all datasets (**Figure S7A**). Univariate and multivariate Cox regression models in both TCGA and CGGA cohorts showed that the TME-score model was an independent prognostic factor (**Figure S8A**). TME-scores were also validated in several external datasets and high TME-scores were found to be prognostic markers for poor survival outcomes (**Figure S9A**).

The ability of the TME-score to predict patients’ response to immune-checkpoint therapy was explored by assigning the GSE35640 cohort patients (melanoma dataset) to different TME-score groups. Patients with high TME-scores exhibited better immunotherapeutic responses (**Figure 6E**). The ROC analyses confirmed that TME-score was a predictive biomarker in patients with melanoma (**Figure 6F**). In another melanoma dataset, GSE78220, patients with high TME-scores exhibited significantly longer OS compared to patients with low TME-scores (**Figure 6G**). High TME-scores also correlated with complete anti-*PD-1* responses (**Figures 6H, I**). The expression patterns of TME-scores in 27 melanoma patients with complete anti-*PD-1* and partial anti-*PD-1* responses and progressive disease are displayed in **Figure S8C**. The ROC analyses confirmed that TME-score was a predictive biomarker in melanoma patients (**Figure 6J**). TME-score was also found to be significantly correlated with two classical immune checkpoint molecules, *LAG3* and *PDCD1LG2* (**Figure S8F**).

To further elucidate the correlation between TME-score and immunotherapy, the potential response to immunotherapy in TCGA based on the TIDE algorithm was evaluated. Patients with high TME-scores exhibited better immunotherapy response compared to those with low TME-scores (**Figure 6C**). Subsequently, responses to anti-*PD-1* and anti-*CTLA-4* therapies were analyzed. The results showed different responses between high and low TME-score groups to both immunotherapies, which a high TME-score exhibited a significant response to anti-*PD-1* immunotherapy while TME-score predicted no response for anti-*CTLA-4* immunotherapy in TCGA (**Figure 6D**). Further, TME-scores were significantly associated with CYT (**Figures 6K, L**). A high TME-score indicated increased expression of GEP (**Figures 6M, N**). TME gene cluster 1 and TME phenotype cluster 1 also showed a high expression of CYT and GEP (**Figures S8D, E**).

### Functional Annotation and Genomic Analysis of TME-Scores

The potential associations between TME-scores and signaling pathways in GO and KEGG pathways based on GSVA were analyzed in TCGA. GO results showed that high TME-scores were significantly associated with immune-related pathways (**Figure S9B**). KEGG analysis showed that a high TME-score was associated with pathways in cancer, apoptosis, and VEGF signaling pathway (**Figure S9C**). These results denoted the complexity of TME and also showed that activated T cells were major components of immune infiltrating cells. GSEA indicated that negative regulation of the immune response and T cell activation were enriched in high TME-scores (**Figure S9D**). Pathways in cancer were associated with high TME-scores as shown in KEGG analysis (**Figure S9E**). A high TME-score indicated the presence of higher overall variants (**Figure S8B**). Additionally, a high TME-score was associated with lower arm SCNA levels and higher chromosome SCNA levels in this study (**Figure S8G**).

### Validation of TME-Scores in the XYNS Cohort

**Figure 7:**
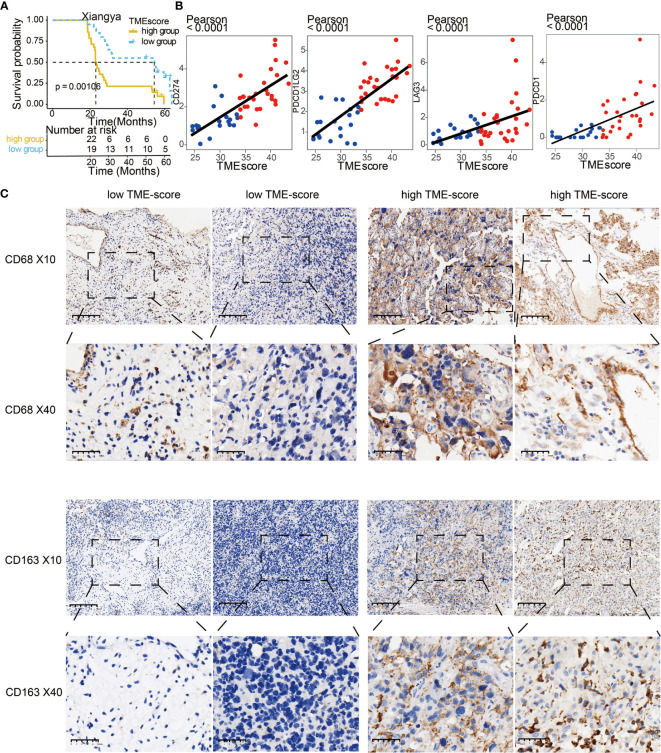
Validation of TME-score in the XYNS cohort. **(A)** Kaplan–Meier curves for high and low TME-score patient groups in Xiangya samples. Log-rank test, P = 0.00106. **(B)** Scatter plots depicting a positive correlation between the TME-score and three classical immune checkpoints, *CD274*, *PDCD1LG2*, *LAG3*, and *PDCD1*. **(C)** Representative images of *CD68* and *CD163* IHC staining based on low and high TME-scores in Xiangya samples.

We validated TME-scores in our samples with sequencing data from Xiangya hospital (**Table S14**). High TME-scores served as prognostic markers for poor clinical outcome in the XYNS cohort (**Figure 7A**). **Figure S10A** shows that the high TME-score exhibited significant higher TME cell infiltration level based on ssGSEA algorithm, and these differences were reported in multiple immune suppressive cell types, including macrophages, mast cells, MDSCs, and Tregs. Gliomas with a high TME-score expressed higher levels of immune checkpoint molecules including *CD274*, *CD276*, *PDCD1LG2*, *LAG3*, *PDCD1*, *TIGIT*, *IDO1*, *CTLA4*, and *TGFB1* (**Figure S10B**). As shown in **Figure 7B**, TME-scores were also found to be positively correlated with four classical immune checkpoints (*CD274*, *PDCD1LG2*, *LAG3*, and PDCD1). Given the critical role of macrophages in the tumor microenvironment, *CD68* and *CD163*, specific M2 macrophage markers, were used for the identification of M2 macrophages in the glioma microenvironment (37). IHC staining showed that high TME-scores exhibited a relatively higher expression of *CD68* and *CD163* compared to low TME-scores (**Figure 7C**), implying a high infiltration of M2 macrophages in the tumor microenvironment with high TME-scores. Besides, IHC staining was also performed for *CD66b* (marker of neutrophil) (38) and *CD117* (marker of mast cell) (39) (**Figure S11**). Likewise, high TME-scores exhibited a relatively higher expression of *CD66b* and *CD117* compared to low TME-scores, implying a high infiltration of neutrophils and mast cells in the tumor microenvironment with high TME-scores.

## Discussion

TME is a complex system that plays an important role in the proliferation and progression of tumor cells. Previous studies have demonstrated that TME also contributes to chemoresistance (7). It is, therefore, considered a novel therapeutic target, especially for immunotherapeutic agents. However, the effects of immunotherapy on gliomas have not been adequately addressed. In this study, we established a TME signature based on prediction of immune infiltrating cells that showed good potential to predict glioma immunotherapy response. The TME signature revealed the immune and stromal statuses, and predicted the survival of patients with glioma. Gene cluster 1 of the TME signature was enriched in genes involved in signaling pathways related to tumor proliferation and progression and was associated with poor survival. Gene cluster 2 had a significant association with immune activation pathways. Immune checkpoints have been shown to facilitate tumor immune evasion (40). In this study, an immunosuppressive microenvironment highly expressing classical immune checkpoints was observed in gene cluster 1.

Based on several consensus computational algorithms, the TME infiltration pattern was estimated and the TME-score for gliomas was established. Most of the 63 differentially expressed genes identified by TME-score, such as *ARHGDIB*, *MYO1F*,and *CD14* have been demonstrated to facilitate tumor proliferation and regulate tumor immune microenvironment in breast (41) and pancreatic (42) cancers, respectively. Analysis of the publicly available datasets and the sequencing data from Xiangya samples indicated that a high TME-score predicted poor survival and an immunosuppressive environment, consistent with the findings in TME phenotypes and TME gene clusters. A high TME-score was also associated with a higher mutation rate of oncogenes, including *TP53* and *PTEN*, while *IDH* mutation, a favorable prognostic marker for gliomas, was detected in low TME-score. Moreover, the TME-score had a high SCNA. In the functional annotation of TME-score in glioma, T cell activation and macrophage activation were significantly correlated with high TME-score. These observations underscore the complexity of biological processes in TME and immune activation that coexist with immune suppression.

The Cox regression analysis showed that the TME-score was associated with high risk in gliomas and several other cancers. Notably, a high TME-score was a favorable marker in melanoma. The patient age, tumor grade, IDH mutation, 1p19q codeletion, and TME-score were all identified as risk factors in glioma patients. Moreover, mesenchymal gliomas had the highest TME-score. A previous study demonstrated that the mesenchymal glioma subtype was associated with an immunosuppressive environment (43), consistent with our results that TME-score could predict an immunosuppressive environment. Notably, TME-score was observed to be significantly involved in the immunological functions of four classical immune suppressive cells including TAMs, MDSCs, Tregs, and CAFs. The IHC staining results proved that M2 macrophages, mast cells, and neutrophils were more infiltrated in tumor microenvironment of patients with high TME-scores. Moreover, high ICP score prominently participated in the regulation of immunomodulators for tumor immunogenicity and antigen presentation capacity. TMB, a diagnostic phenotype with more malignancy of cancer and better immunotherapy response, was more significantly correlated with high TME-score (44). High TME-score was also detected with higher Intratumor Heterogeneity, a diagnostic phenotype with more malignancy of cancer (45). Additionally, high TME-score had the distinct biological characteristics regarding stroma signatures such as TGF-beta response and leukocyte fraction compared with low TME-score, and these stroma signatures have previously been proved to facilitate the immune escape of cancer (46). Therapeutic inhibitors that block the *PD-1*/*PD-L1* pathway have been reported to enhance immunotherapy responses in multiple cancers (32, 47–51). So far, anti-*PD-1* therapy has not been effective in glioma cohorts, and one phase 3 trial failed to show that *PD-1* inhibition confers a survival benefit in patients with recurrent glioblastoma (52). Therefore, we examined the impact of TME-score on anti-*PD-1* therapy based on two melanoma cohorts, GSE35640, and GSE78220. Patients with high TME-score were more likely to benefit from anti-*PD-1* therapy, demonstrating the different immune infiltrating microenvironment between gliomas and melanoma. Using the TIDE algorithm, high TME-scores correlated with good response to immunotherapies such as anti-*PD-1* and anti-*CTLA-4*. Thus, we hypothesized that the TME-score may potentially serve as a sensitive marker for predicting glioma patients’ response to anti-*PD-1* therapy.

A positive correlation was observed between TME-score, GEP, and CYT. GEP-induced CYT enhanced the anti-tumor activity of the adoptive transfer of T cells. These results may appear contradictory to the perilous role of high TME-score in gliomas. However, this could be attributed to TME complexity, where activated T cells coexist with multiple immunosuppressive infiltrating cells. Previous studies have shown that T cell–infiltrated tumors have an optimal response to therapies targeting the immune system inhibitory mechanisms (53). The high TME-score was positively involved in T cell activity and indicated a better response to anti-*PD-1* therapy.

Although many studies have established prognostic models based on several immune-related signature genes, they were based on small samples and only utilized a small fraction of TME (**Table S9**). In this study, we developed a TME-score based on several signature genes that enabled us to comprehensively explore the infiltration characteristics of the TME cells in individual glioma patients. Thus, the TME-score would help study the immune phenotype of tumors thereby improving clinical management. The performance of the TME-score was consistent with findings from TME clusters and TME gene clusters. Further analysis showed that the TME-score could assess patients’ clinicopathological features, including the immune infiltration pattern, tumor stage, age, molecular subtypes, and genetic variations. TME-score showed good potential as an independent prognostic biomarker in predicting patient survival. Currently, there are multiple ongoing clinical trials on immunotherapy targeting *PD-1*; however, they have not demonstrated promising results so far (**Table S10**). Therefore, the TME-score established here would help evaluate clinical response to anti-*PD-1* therapy and promote the development of effective immunotherapy strategies.

## Data Availability Statement

The original contributions presented in the study are included in the article/**Supplementary Material**. Further inquiries can be directed to the corresponding authors.

## Author Contributions

HZ, QC, NZ, ZW, ZD, XZ, and ZL designed and drafted the manuscript. HZ and QC wrote figure legends and revised the article. QC and NZ conducted the data analysis. All authors contributed to the article and approved the submitted version.

## Funding

This work was supported by the National Natural Science Foundation of China (NO. 82073893, 81472693, 81873635, and 81703622), China Postdoctoral Science Foundation (NO. 2018M633002), Hunan Provincial Natural Science Foundation of China (NO. 2018SK2101, 2018JJ3838), Hunan Provincial Health Committee Foundation of China (C2019186). Xiangya Hospital Central South University postdoctoral foundation.

## Conflict of Interest

The authors declare that the research was conducted in the absence of any commercial or financial relationships that could be construed as a potential conflict of interest.

## Publisher’s Note

All claims expressed in this article are solely those of the authors and do not necessarily represent those of their affiliated organizations, or those of the publisher, the editors and the reviewers. Any product that may be evaluated in this article, or claim that may be made by its manufacturer, is not guaranteed or endorsed by the publisher.

## Abbreviations

WHO, World Health Organization; LGG, low-grade glioma; GBM, glioblastoma; TME, tumor microenvironment; TAM, tumor-associated macrophages; TANs, tumor-associated neutrophils; CAFs, cancer-associated fibroblasts; MDSCs, myeloid-derived suppressor cells; Tregs, regulatory T cells; GEO, Gene-Expression Omnibus; CGGA, Chinese Glioma Genome Atlas; TCGA, The Cancer Genome Atlas; FPKM, fragments per kilobase million; TPM, transcripts per kilobase million; PAM, Partitioning Around Medoid; DEGs, differentially expressed genes; PCA, principal component analysis; GSEA, gene set enrichment analysis; GSVA, gene set variation analysis; TIDE, Tumor Immune Dysfunction and Exclusion; ROC, receiver operating characteristic; AUC, area under the curve; GEP, gene expression profile; CYT, cytotoxic activity; ICB, immune checkpoint blockade; SCNAs, somatic copy number alterations; IHC, immunohistochemistry.
