# Supplementary material for: Immune Infiltrating Cells-Derived Risk Signature Based on Large-scale Analysis Defines Immune Landscape and Predicts Immunotherapy Responses in Glioma Tumor Microenvironment
Source: Front Immunol. 2021 Aug 13;12:691811. doi: 10.3389/fimmu.2021.691811 (PMC8418124; doi:10.3389/fimmu.2021.691811)
Supplement: Supplementary file 2 [file DataSheet_2.docx]

**Figure Legends**


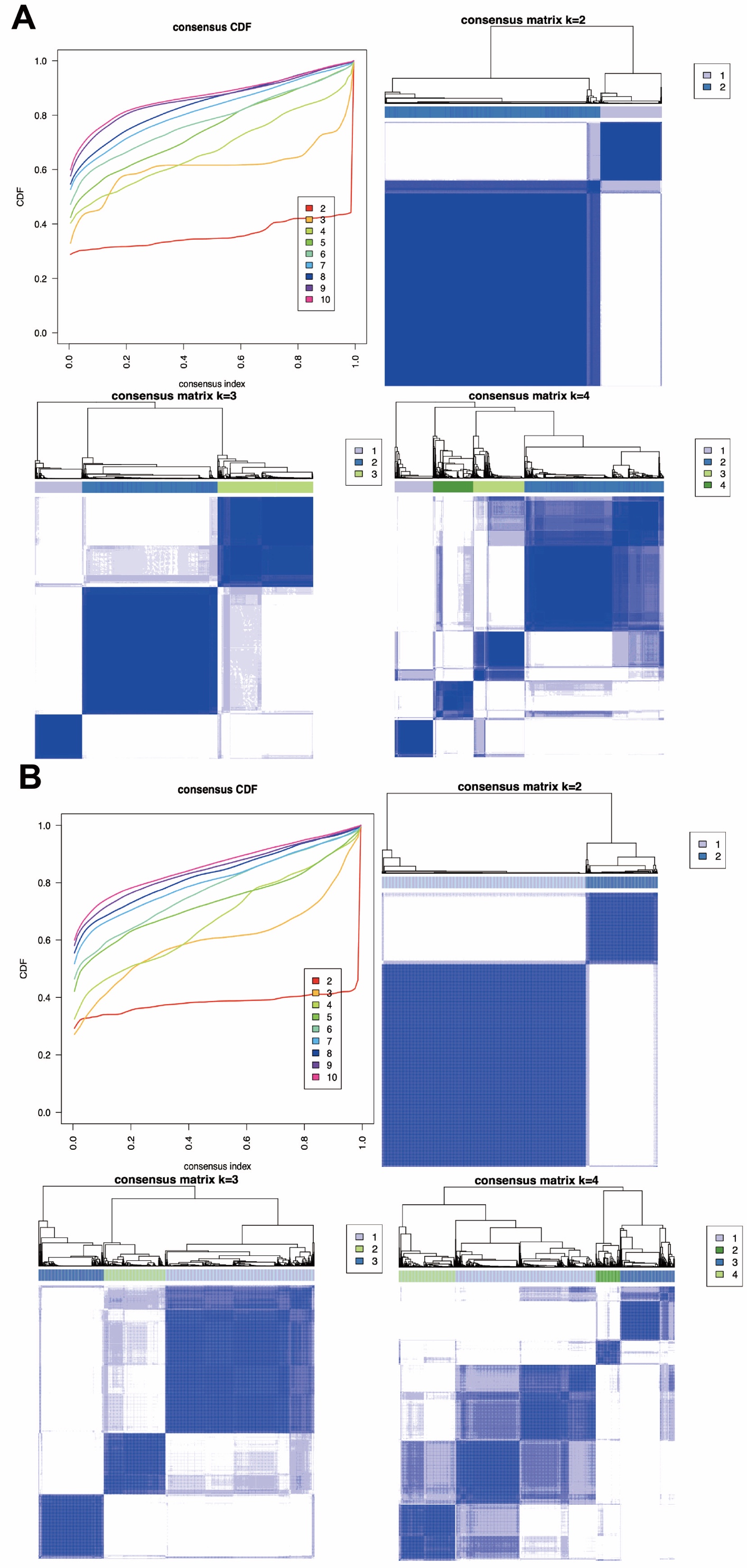


**Figure S1.** (**A**) Consensus clustering cumulative distribution function (CDF) for k=2 to 10 in meta-cohort. Consensus matrices of meta-cohort for each k (k = 2–4), displaying clustering stability using 1000 iterations of hierarchical clustering. (**B**) Consensus clustering cumulative distribution function (CDF) for k=2 to 10 in TCGA. Consensus matrices of TCGA for each k (k = 2–4), displaying clustering stability using 1000 iterations of hierarchical clustering.


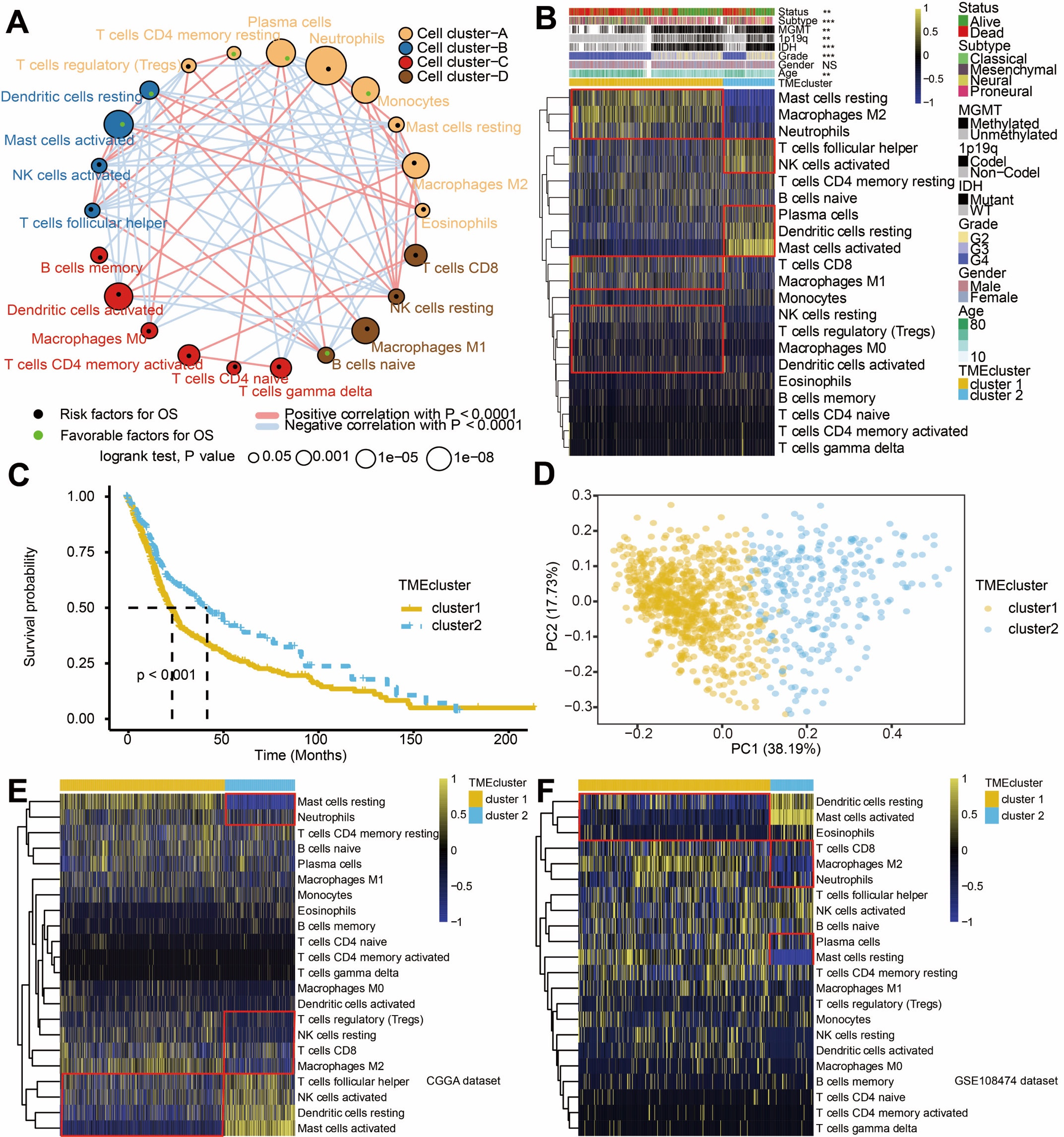


**Figure S2. TME landscape in gliomas and subtype characteristics in TCGA.** (**A**) Cellular interaction of TME cell types. Cell cluster A, orange; cell cluster B, blue; cell cluster C, red; cell cluster D, brown. Each cell’s size represents the survival impact of each TME cell type; calculations used the formula log10 (log-rank test P values indicated). Favorable factors for overall survival are indicated in green, and risk factors indicated in black. The lines connecting TME cells represent cellular interactions. The positive correlation is indicated in red and negative correlation in blue. (**B**) Unsupervised clustering of TME cells for 1027 patients in TCGA. Molecular subtype, survival status, Patient age, sex, MGMT status, 1p19q status, IDH status, tumor grade, and TME clusters are shown as patient annotations. The statistical difference of two TME clusters was compared through the Kruskal–Wallis test. *, P < 0.05; **, P < 0.01; ***, P < 0.001. (**C**) Kaplan-Meier curves for two TME groups of 1027 patients in TCGA. Log-rank test, P < 0.001. (**D**) PCA separated the two TME clusters. (**E**) Unsupervised clustering of TME cells for glioma patients in the CGGA cohort. Hierarchical clustering was performed with Euclidean distance and Ward linkage. Rows represent TME cells and columns represent samples. (**F**) Unsupervised clustering of TME cells for glioma patients in the GSE108474 cohort. Hierarchical clustering was performed with Euclidean distance and Ward linkage. Rows represent TME cells, and columns represent samples.


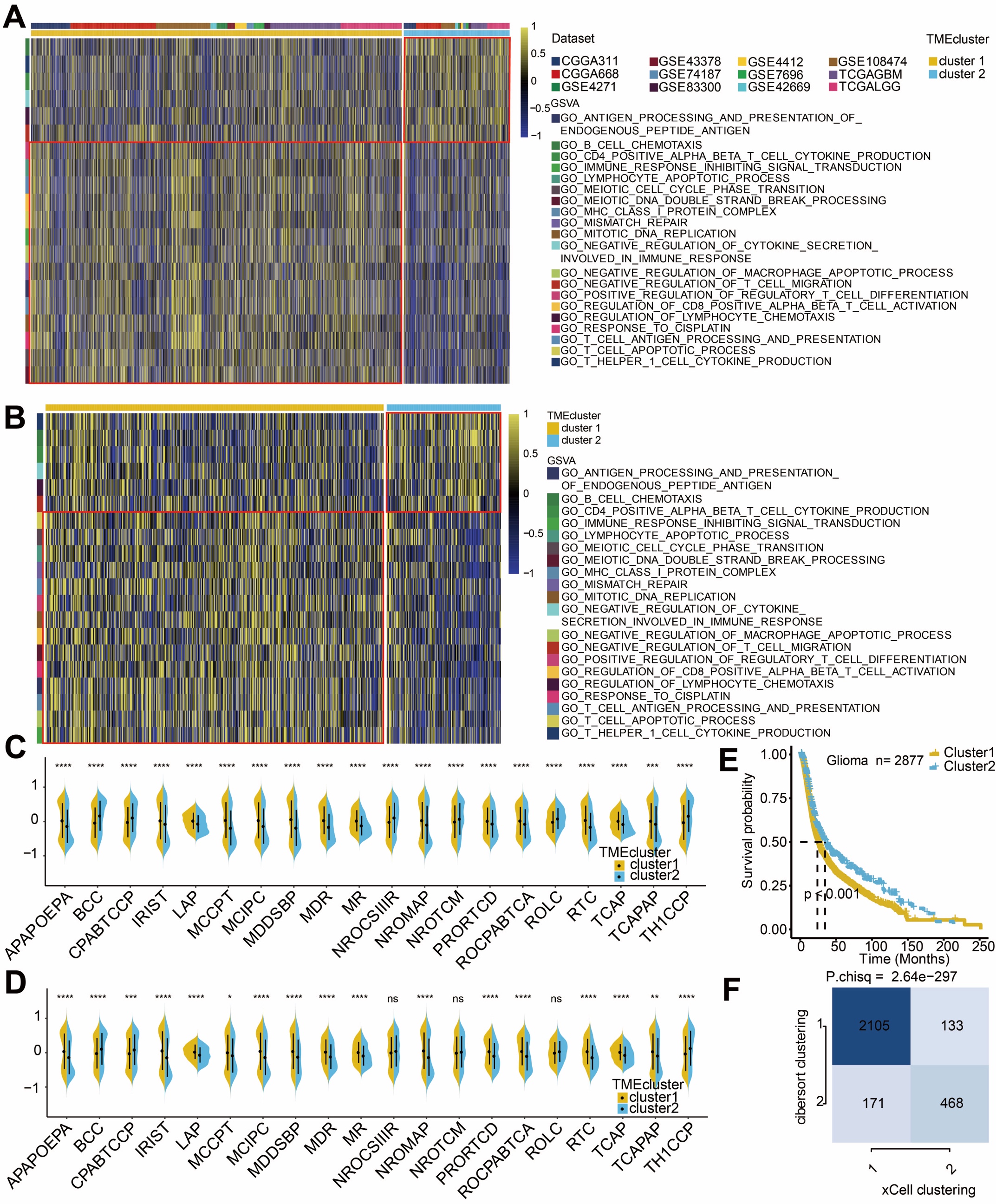


**Figure S3. Immune-related functional annotation of TME clusters.** (**A**) GSVA of TME clusters based on meta-cohort in GO. (**B**) GSVA of TME clusters based on TCGA in GO. (**C**) Expression difference of GSVA resulting in TME clusters in the meta-cohort. (**D**) Expression difference of GSVA resulting in TME clusters in TCGA. (**E**) Kaplan–Meier curves for TME clusters constructed via xCell algorithm in the meta-cohort. Log-rank test, P < 0.001. (**F**) Contingency table reveals the consistency between TME clusters identified via xCell algorithm and TME clusters identified via CIBERSORT algorithm.


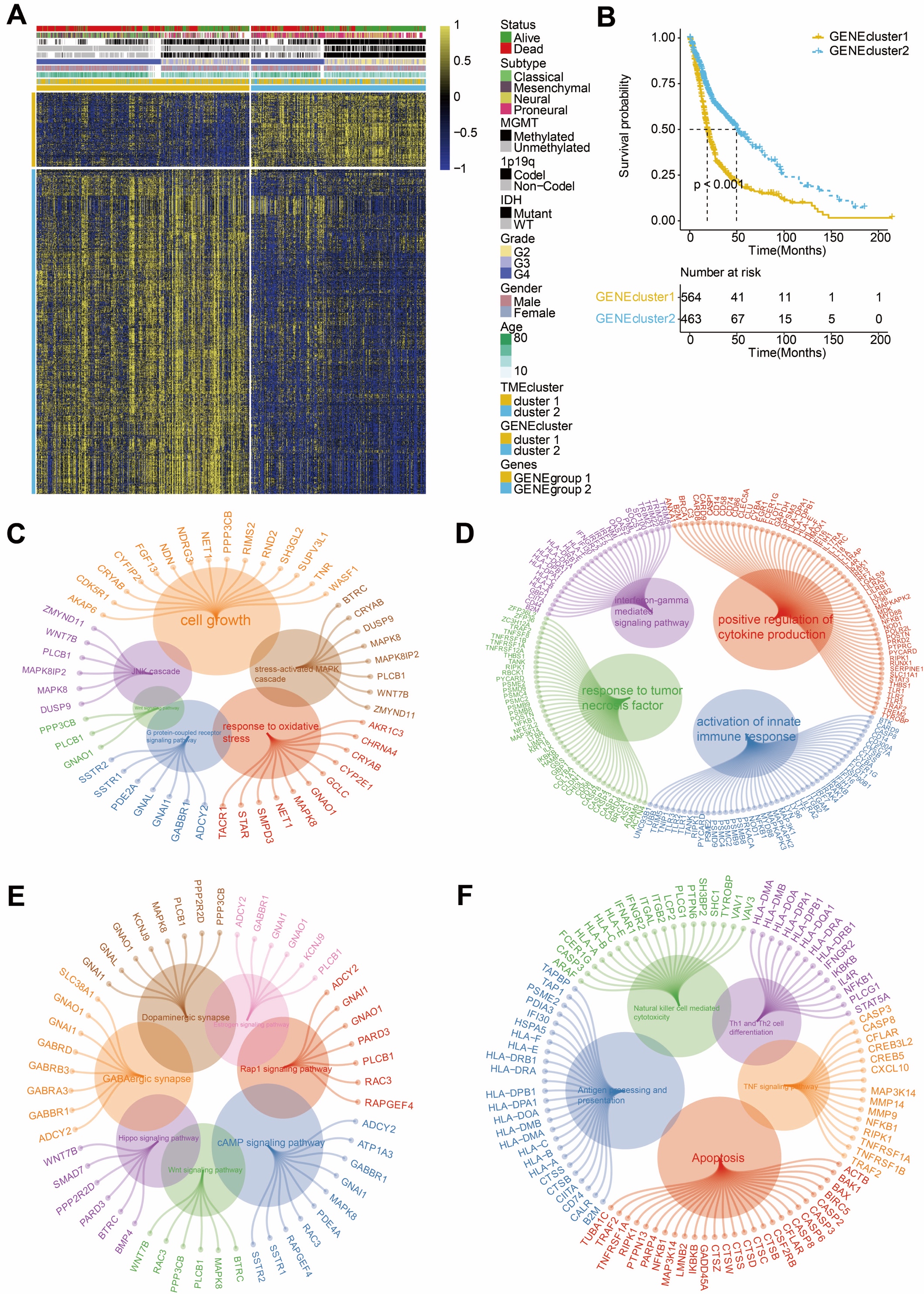


**Figure S4.** **Construction of TME signatures and functional annotation.** (**A**) Unsupervised analysis and hierarchical clustering of common DEGs based on expression data of gliomas derived from TCGA to classify patients into two groups: Gene clusters 1 and 2. The molecular subtype, survival status, patient age, MGMT status, 1p19q status, IDH status, tumor grade, patient gender, and TME clusters are shown as patient annotations. (**B**) Kaplan–Meier curves for two TME gene clusters in TCGA. Log-rank test, P < 0.001. (**C**) GO enrichment analysis of relevant signature genes in TME gene cluster 1. (**D**) GO enrichment analysis of relevant signature genes in TME gene cluster 2. (**E**) KEGG enrichment analysis of TME-relevant signature genes in TME gene cluster 1. (**F**) KEGG enrichment analysis of TME relevant signature genes in TME gene cluster 2.


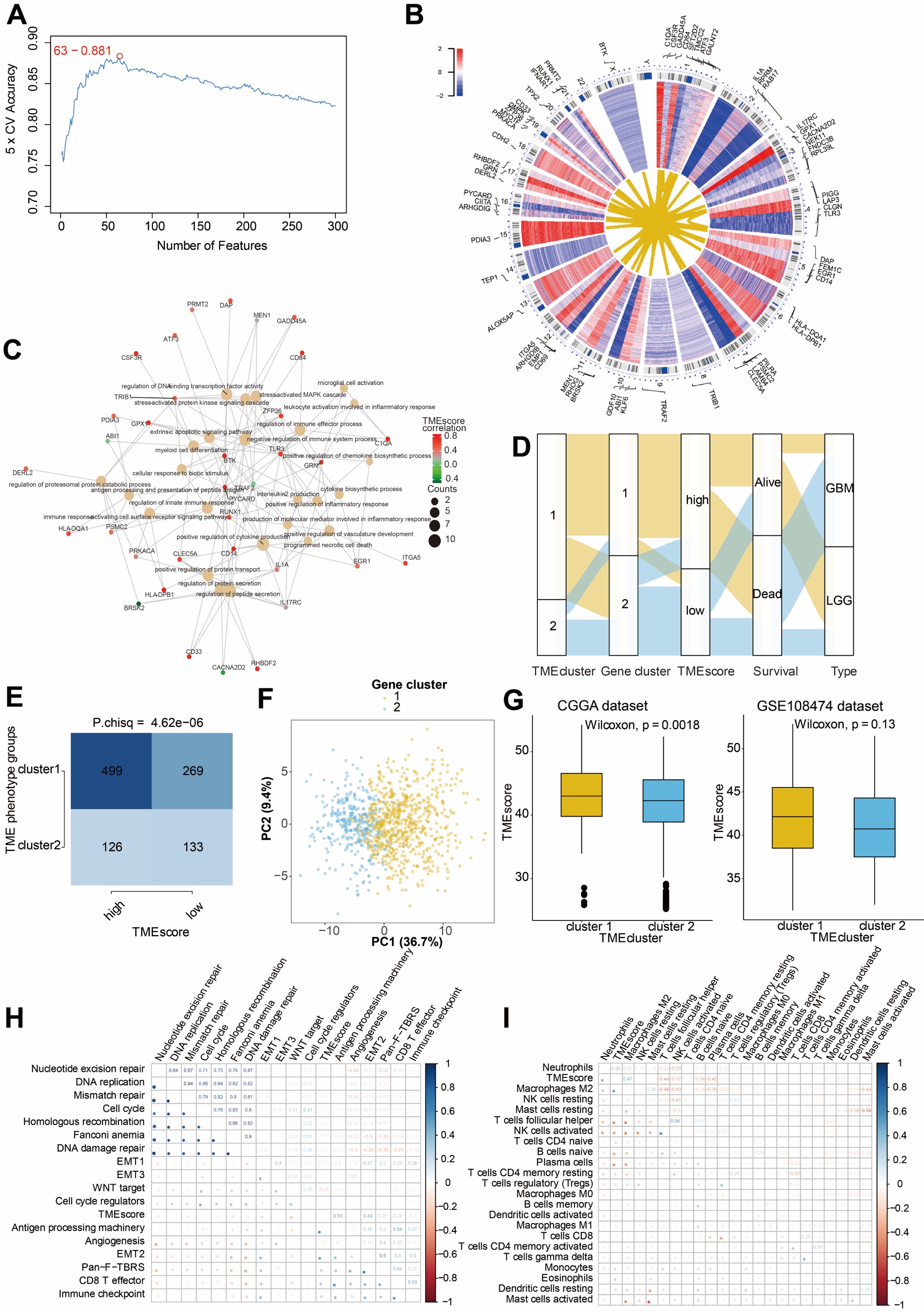


**Figure S5. 63 TME signature genes.** (**A**) SVM-RFE algorithms confirming 63 most representative DEGs to be optimal for the unsupervised hierarchical cluster analysis. (**B**) Heatmap illustrating the distribution of the 63 DEGs in human chromosomes. Expression values are z-transformed and are colored red for high expression and blue for low expression, as illustrated in the scale bar. (**C**) Visualization of the potential regulatory network of 63 TME signature genes. (**D**) Alluvial diagram showing association among TME clusters, TME gene clusters, TME-score, patient survival, and tumor grade. (**E**) Contingency table reveals the consistency between TME clusters and TME-score groups. (**F**) PCA of 63 DEGs separated the two TME gene clusters. (**G**) Distribution of TME-score of CGGA and GSE108474 cohorts in TME cluster 1 and TME cluster 2. Differences between the two groups were compared by the Kruskal-Wallis test. (**H**) Correlations between TME-score and known gene signatures linked to epithelial-mesenchymal transition (EMT), immune checkpoint, mismatch repair, and immune activation in TCGA. (**I**) Correlations between TME-score and immune infiltrating cells in TCGA.


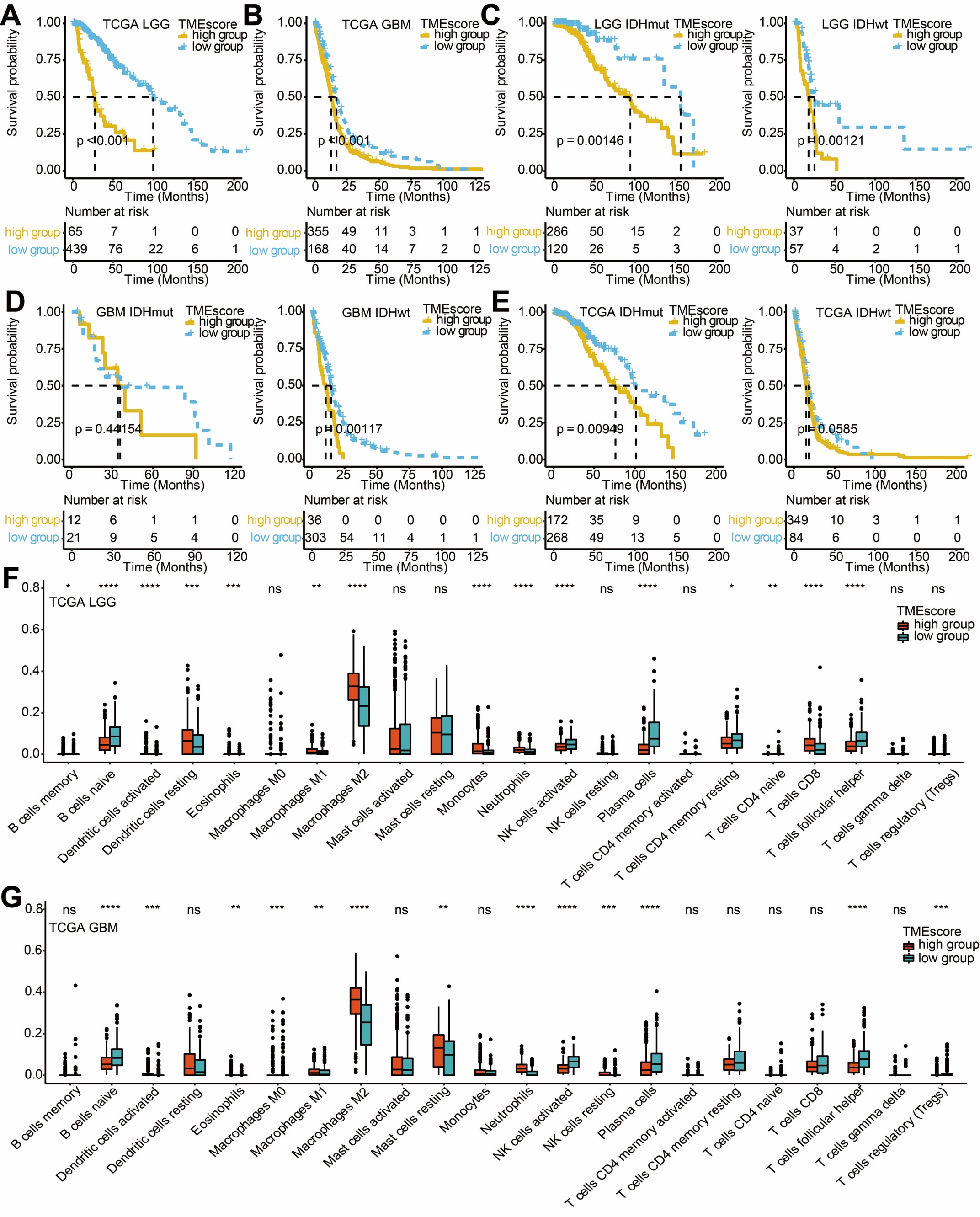


**Figure S6.** (**A**) Kaplan-Meier curves for high and low TME-score patient groups in TCGA LGG samples. Log-rank test, P < 0.001. (**B**) Kaplan-Meier curves for high and low TME-score patient groups in TCGA GBM samples. Log-rank test, P < 0.001. (**C**) Kaplan-Meier curves for high and low TME-score patient groups in TCGA LGG IDHmut and TCGA LGG IDHwt samples. (**D**) Kaplan-Meier curves for high and low TME-score patient groups in TCGA GBM IDHmut and TCGA GBM IDHwt samples. (**E**) Kaplan-Meier curves for high and low TME-score patient groups in TCGA pan-gliomas IDHmut and TCGA pan-gliomas IDHwt samples. (**F**) Fraction of TME cells in TME-score in TCGA LGG samples. Within each group, the scattered dots represent TME cell expression values. (**G**) Fraction of TME cells in TME-score in TCGA GBM samples. Within each group, the scattered dots represent TME cell expression values. The thick line represents the median value. The bottom and top of the boxes are the 25th and 75th percentiles (interquartile range). The whiskers encompass 1.5 times the interquartile range. The statistical difference of two TME clusters was compared by the Kruskal–Wallis test. *, P < 0.05; **, P < 0.01; ***, P < 0.001; ****, P < 0.0001.


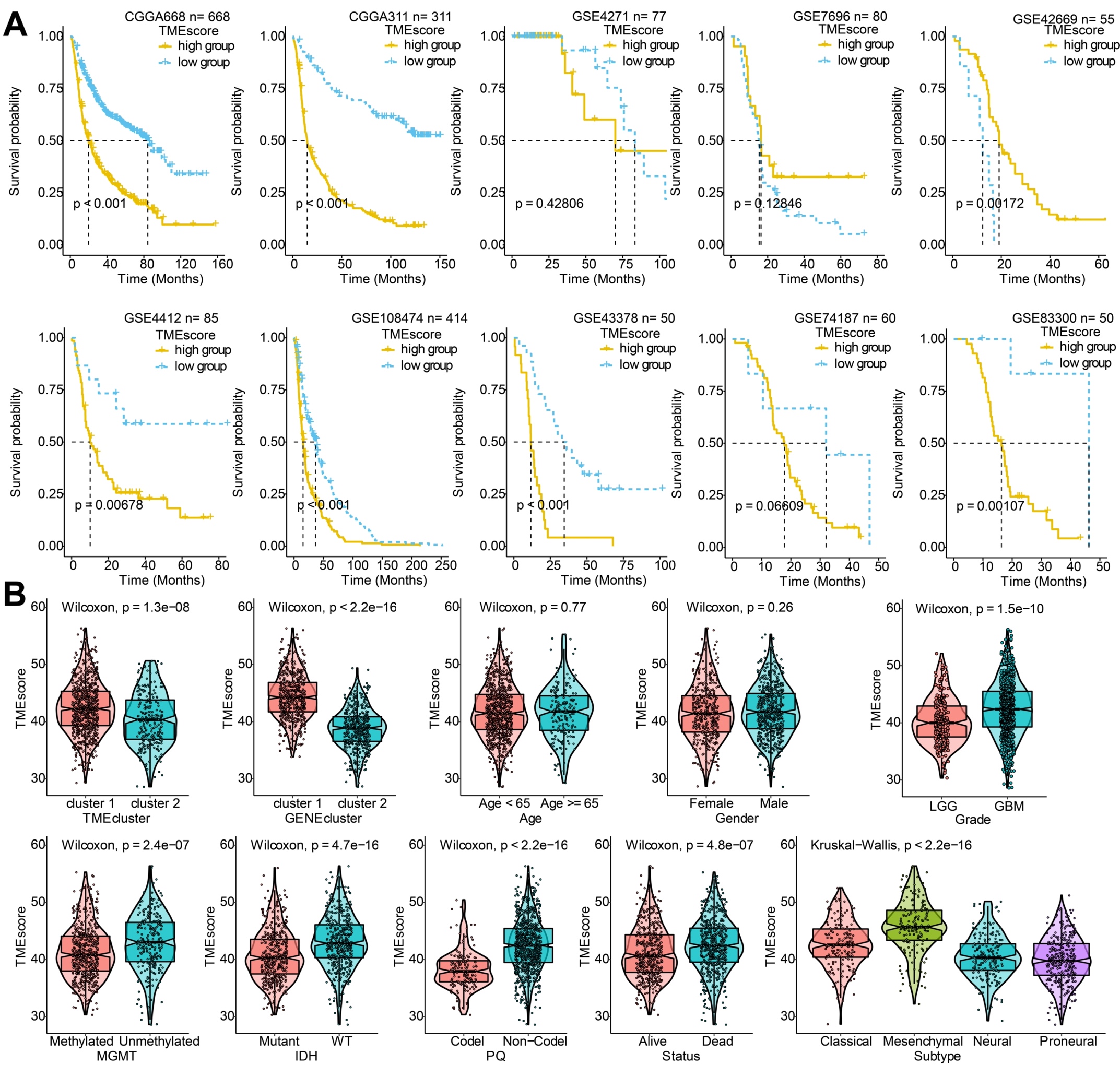


**Figure S7.** (**A**) Kaplan–Meier curves for the high and low TME-score patient groups in the 10 included cohorts, CGGA311, CGGA668, GSE4271, GSE4412, GSE7696, GSE42669, GSE43378, GSE74187, GSE83300, and GSE108474. (**B**) Expression pattern of TME-scores in TME clusters, TME gene clusters, patient age, patient gender, tumor grade, MGMT status, IDH status, 1p19q status, and glioma molecular subtypes.


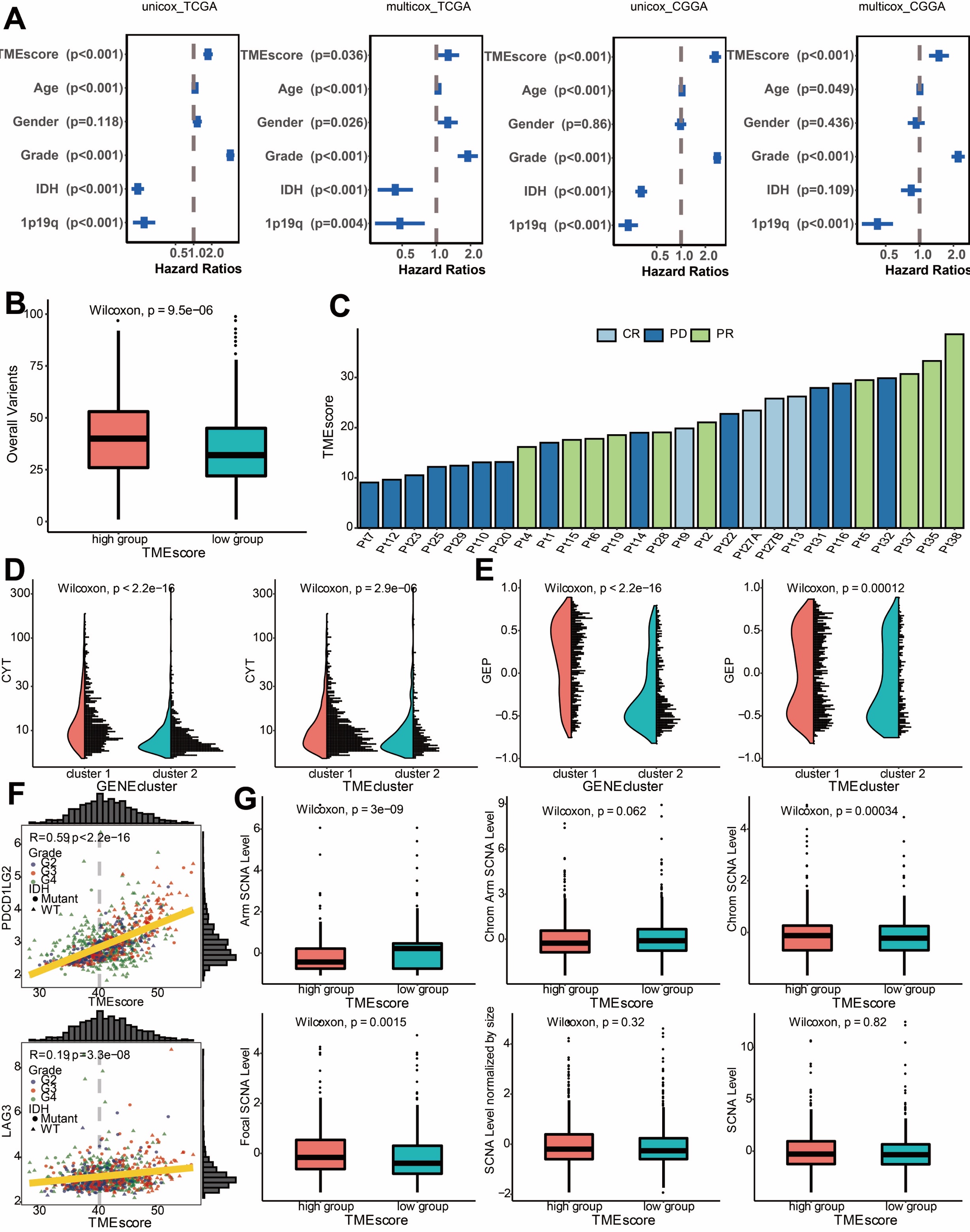


**Figure S8.** (**A**) Univariate multivariate cox regression analyses to estimate clinical prognostic factors, including TME-score in TCGA and CGGA, respectively. The length of the horizontal line represents a 95% confidence interval for each group. The vertical dotted line represents the hazard ratio (HR) in all patients. (**B**) Expression difference of overall variants in high and low TME-scores. (**C**) Visualization of TME-score in each patient with different responses to anti-PD-1 therapy in the GSE78220 cohort. (**D**) Expression difference of CYT in TME gene clusters and TME clusters. The differences between groups were compared by the Wilcoxon test (Wilcoxon, P<0.001). (**E**) GEP expression difference in TME gene clusters and TME clusters. The differences between groups were compared by the Wilcoxon test (Wilcoxon, P<0.001). (**F**) Scatter plots depicting the positive correlation between TME-score and PDCD1LG2. Pearson correlation coefficient R=0.59. Scatter plots depicting a positive correlation between TME-score and LAG3. Pearson correlation coefficient R=0.19. (**G**) Expression difference of different SCNA levels in high and low TME-scores.


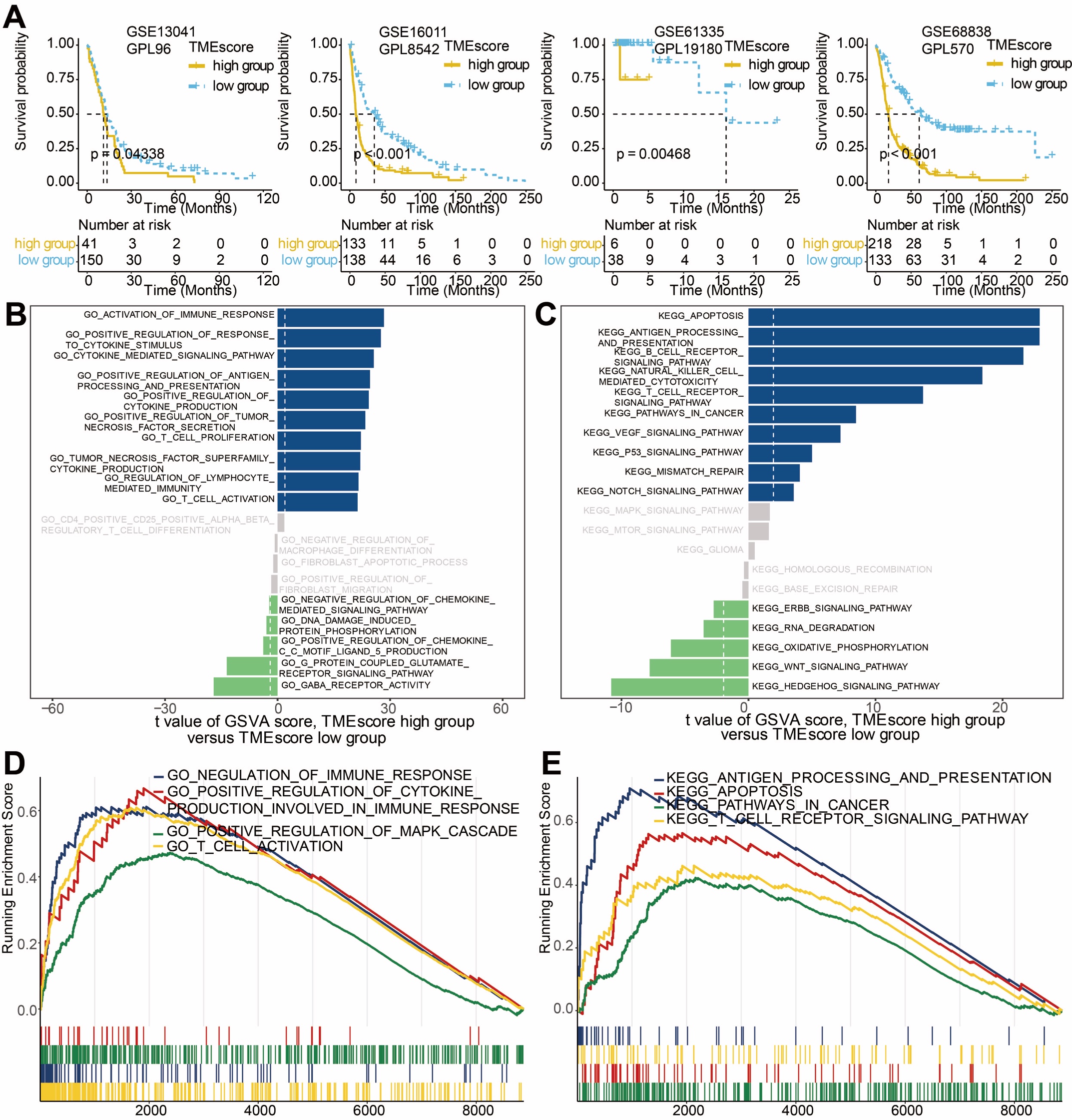


**Figure S9. Immune-related functional annotation of TME-score.** (**A**) Kaplan–Meier curves for high and low TME-score patient groups in external datasets, including GSE13041, GSE16011, GSE61335, and GSE68838. (**B**) GSVA of TME-score based on TCGA in GO. (**C**) GSVA of TME-score based on TCGA in KEGG. (**D**) GSEA of TME-score based on TCGA in GO. (**E**) GSEA of TME-score based on TCGA in KEGG.


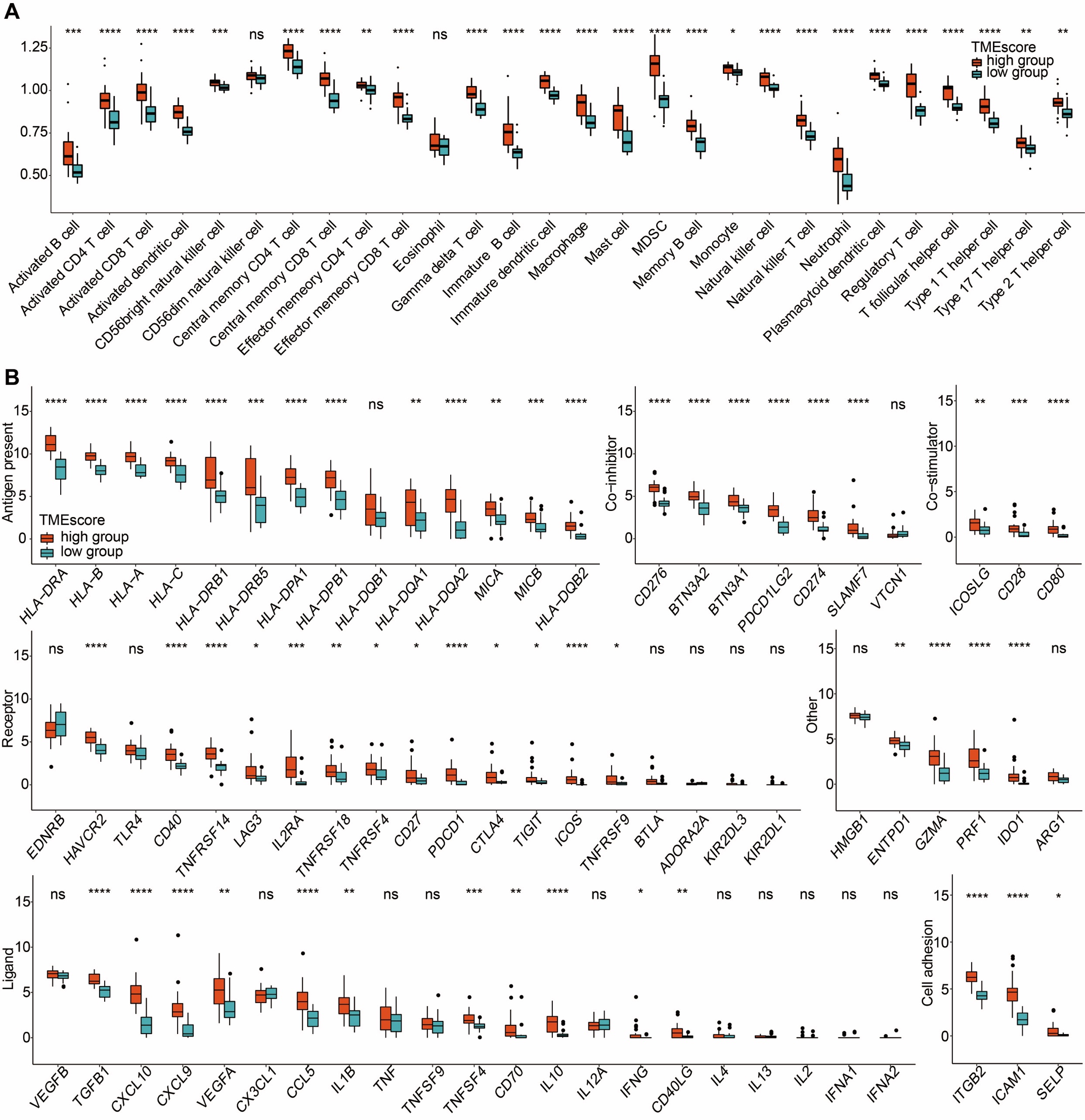


**Figure S10. Immune characteristics of TME score in Xiangya cohort.** (**A**) The fraction of immune infiltrating cells in TME score in the Xiangya cohort. (**B**) The fraction of seven types of immune checkpoints in TME score in the Xiangya cohort. Within each group, the scattered dots represent immune checkpoint expression values. The thick line represents the median value. The bottom and top of the boxes are the 25th and 75th percentiles (interquartile range). The whiskers encompass 1.5 times the interquartile range. The statistical difference of two TME clusters was compared by the Kruskal–Wallis test. *, P < 0.05; **, P < 0.01; ***, P < 0.001; ****, P < 0.0001.


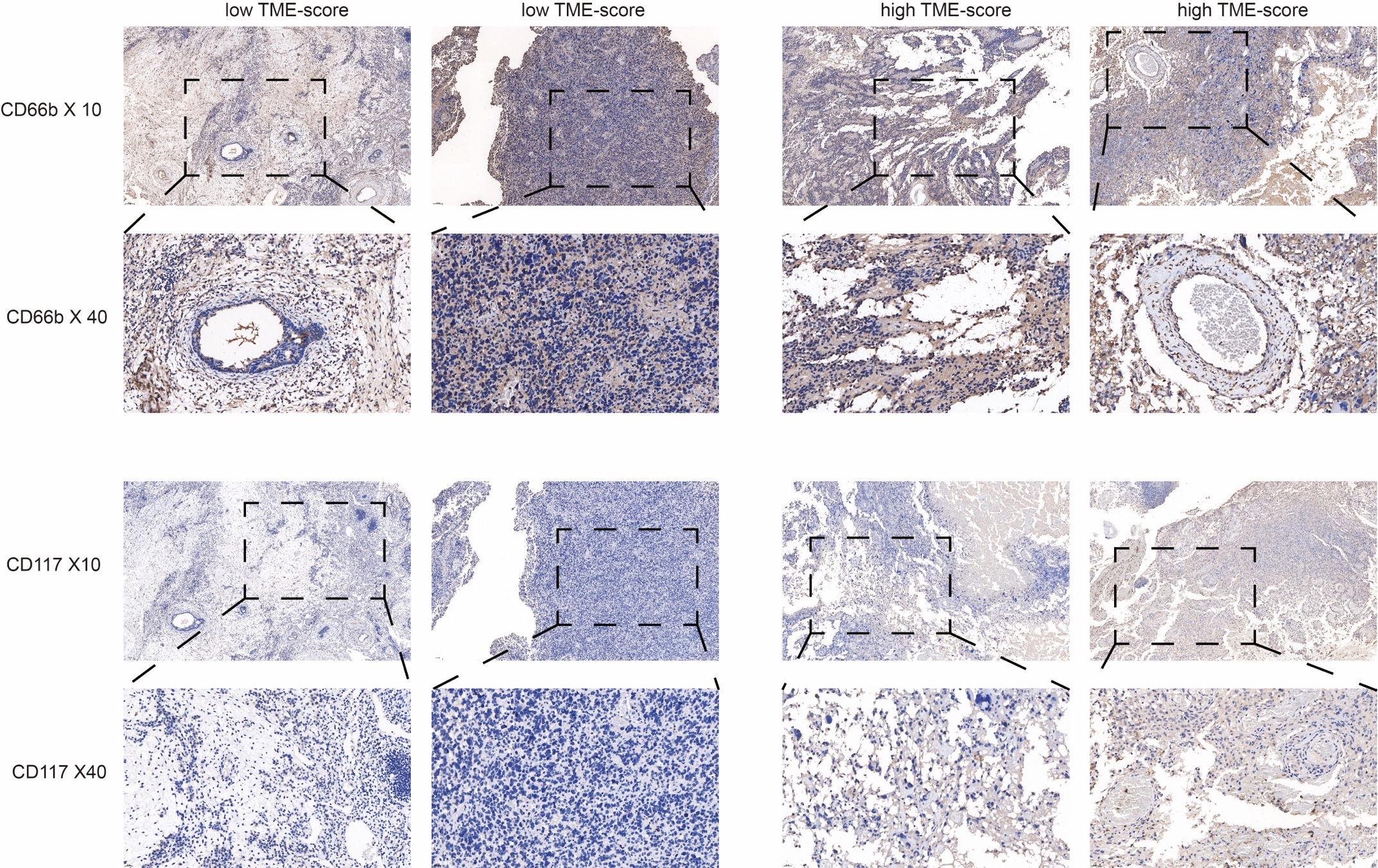


**Figure S11.** Representative images of *CD66b* and *CD117* IHC staining based on low and high TME-scores in Xiangya samples.

**Table legends**

Table S1: Basic information of 12 included cohorts

Table S2: Relative fractions of tumor microenvironment cells of 2877 glioma patients with overall survival information

Table S3: Hazard ratios of tumor microenvironment cells in gliomas

Table S4: Cellular interaction between tumor microenvironment cells

Table S5: 1312 Differentially expressed genes in TCGA

Table S6: The distribution of TME clusters in TCGA, CGGA, and GSE108474 cohorts

Table S7: Sixty-three representative TME signature genes

Table S8: TME-score in the 12 cohorts

Table S9: Previous immune-related prognostic models in gliomas

Table S10: Recent clinical trials of immunotherapy targeting PD-1 in gliomas

Table S11: Significantly different CNV regions compared between high and low TME-score groups

Table S12: Significantly different mutated genes compared between high and low TME-score groups

Table S13: Expression difference of 63 genes between IDHmut and IDHwt

Table S14: Clinical characteristics of Xiangya cohort
